# IL-2 imprints human naive B cell fate towards plasma cell through ERK/ELK1-mediated BACH2 repression

**DOI:** 10.1038/s41467-017-01475-7

**Published:** 2017-11-13

**Authors:** Nicolas Hipp, Hannah Symington, Cédric Pastoret, Gersende Caron, Céline Monvoisin, Karin Tarte, Thierry Fest, Céline Delaloy

**Affiliations:** 1 UMR U1236, Université de Rennes 1, INSERM, Etablissement Français du Sang (EFS) de Bretagne, Equipe labellisée Ligue contre le Cancer, Labex IGO, 2 Av du Pr Léon Bernard, 35043 Rennes, France; 2 Laboratoire d’Hématologie, Centre Hospitalier Universitaire (CHU) Rennes, 2 rue Henri Le Guilloux, 35033 Rennes Cedex 9, France; 3 Laboratoire d’Immunologie, Thérapie Cellulaire et Hématopoïèse (ITeCH), Centre Hospitalier Universitaire (CHU) Rennes, 2 rue Henri Le Guilloux, 35033 Rennes Cedex 9, France

## Abstract

Plasma cell differentiation is a tightly regulated process that requires appropriate T cell helps to reach the induction threshold. To further understand mechanisms by which T cell inputs regulate B cell fate decision, we investigate the minimal IL-2 stimulation for triggering human plasma cell differentiation in vitro. Here we show that the timed repression of BACH2 through IL-2-mediated ERK/ELK1 signalling pathway directs plasma cell lineage commitment. Enforced BACH2 repression in activated B cells unlocks the plasma cell transcriptional program and induces their differentiation into immunoglobulin M-secreting cells. RNA-seq and ChIP-seq results further identify BACH2 target genes involved in this process. An active regulatory region within the *BACH2* super-enhancer, under ELK1 control and differentially regulated upon B-cell activation and cellular divisions, helps integrate IL-2 signal. Our study thus provides insights into the temporal regulation of BACH2 and its targets for controlling the differentiation of human naive B cells.

## Abstract (web summary)

T cells help B cells to differentiate into antibody-producing plasma cells. Here the authors show that T cells produce interleukin-2 to activate ERK/ELK1 and suppress BACH2 expression by modulating the BACH2 super-enhancer, thereby altering BACH2 downstream transcription programs for plasma cell differentiation.

## Introduction

A well-characterised gene regulatory network governs the transition of a naive B cell precursor to either a plasma cell or a memory B cell within secondary lymphoid organs^1,2^. Following antigen-priming B cells enter into long-lasting interactions with antigen-specific CD4+ helper T cells at the border of T and B zones^3^. These precursors of T follicular helper cells provide a plethora of signals, costimulatory molecules and cytokines, that can promote B-cell survival, proliferation, and B cell commitment into plasma cells, germinal centre (GC) cells or memory B cells^4^. Temporal dynamic of cell signalling pathways regulating the transcription factor network and influencing B cell fate decision still remains to be investigated. It is suggested that transcriptional repression dominates the program leading to plasma cell differentiation^5–7^. Indeed, B cell transcription factors are collectively involved in repressing *PRDM1*/BLIMP1 expression, the multitasking transcription factor in plasma cells^8,9^. The downregulation of PAX5 early after B-cell activation occurs independent of BLIMP1, suggesting that PAX5 could be the trigger of plasma cell differentiation^10^. IRF4 and IRF8 are proposed to antagonise each other for regulating the initial bifurcation in activated B cell fate^11^. Alternatively, evidences showed that the level of BACH2 instructs B cells to undergo differentiation into either plasma cells or memory cells^6,12,13^. BACH2 binds to MARE motifs and cooperates with BCL6 on *PRDM1* promoter^14,15^. However, additional targets of BACH2 beyond *PRDM1* during the transition from activated B cells to plasma cells must be elucidated. Moreover, the precise mechanisms regulating *BACH2* expression in activated B cells remain unknown despite the description of a super-enhancer in the *BACH2* locus^16,17^.

Difficulties to study signal integration during B cell terminal differentiation originate from heterogeneous and asynchronous cellular responses to differentiation-inducing stimuli^18–20^. Indeed, antigen affinity and the various co-stimuli of the complex microenvironment that are integrated in a spatial and temporal dynamic manner affect the differentiation process in cascade. In this context, obtaining sufficient number of primary activated B cells, which are rare and transient in vivo, is problematic. Many aspects of human plasma cell differentiation are recapitulated in a primary culture system combining B-cell receptor (BCR) signal, Toll like receptor activation and T cell helps (CD40L and cytokines)^21,22^. Naive B cells undergo class-switch recombination (CSR) and give rise to plasma cells under these defined conditions. T cell-produced interleukin-2 (IL-2) is one early minimal input required for eliciting differentiation in this model system, independently from proliferation and survival effects^21^. The underlying mechanism involves the extracellular signal-regulated kinase (ERK1/2) signalling pathway. Accordingly, mice models have confirmed the critical role of interleukins and ERK signalling in the initiation of plasma cell differentiation^23^. ERK signalling pathway was shown to be involved in immune cell cycle progression and survival^24^, but its function in terminal differentiation is still controversial, as opposing effects of BCR-induced ERK activation for plasma cell differentiation have both been described in vitro^25,26^.

Here we study the function of IL-2-induced ERK signalling for plasma cell lineage commitment. We take advantage of a controlled and well-defined in vitro model of the human plasma cell differentiation^21,22^ to catch the transient states of B-cell activation and to follow single-cell destiny. We establish that IL-2-ERK-ELK1 signalling pathway overcomes the repressive forces that block plasma cell differentiation. We identify BACH2 and its target genes as major effectors of the IL-2-ERK-ELK1 signalling pathway for controlling B cell terminal differentiation. Our results suggest a molecular switch of ELK1 acting within the *BACH2* super-enhancer to fine-tune *BACH2* expression. In conclusion, our data add to the understanding of *BACH2* temporal regulation and function in the process of human B-cell activation with important implications for plasma cell differentiation efficiency.

## Results

### Heterogeneity of B cell response to IL-2 stimulation

**Fig. 1:**
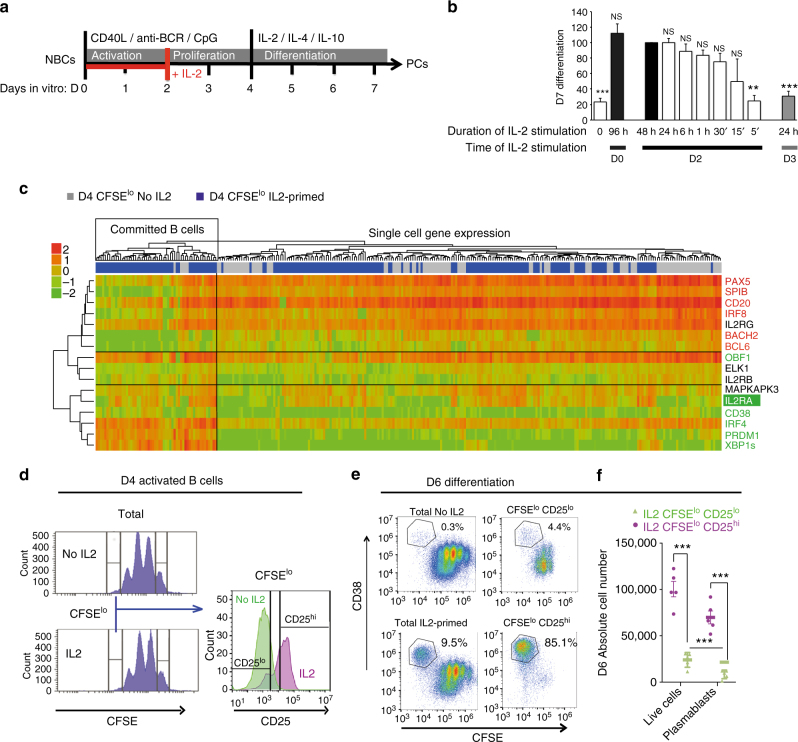
A minimal early and transient IL-2 signal confers plasma cell differentiation ability to human naive B cells. **a** Experimental design to differentiate primary naive B cells (NBCs) into plasma cells (PCs). Activation of peripheral blood mature naive B cells was initiated by BCR cross-linking (anti-BCR), CpG and CD40L during 4 days followed by cell washing and induction of terminal differentiation into plasma cells with IL-2, IL-4 and IL-10 over 3 days. Antigen-primed naive B cells have 2-day window (in red) for acquisition of early T cell help in the form of IL-2 which is required to recruit B cells into the plasma cell differentiation program. **b** Kinetic experiments of IL-2 stimulation to determine the time window (in day, D) and the minimal duration (in hours, h) for B cell to acquire differentiation ability. IL-2 was added in the medium at the indicated day. Neutralising antibodies against IL-2 or IL2 receptors were used to block IL-2 signalling and isotype antibodies were used as controls. Results are the mean of plasma cell numbers with the s.d. quantified at D7 by flow cytometry and fixed to 100 for the condition corresponding to 48 h of IL-2 stimulation (black bar) to normalise between experiments (*n* = 5). **c** Single-cell QRT-PCR analysis in the D4-CFSE^lo^ subset of cells that were primed with IL-2 (in blue, *n* = 175) or not (in grey, *n* = 110) at D2. The heatmap shows the spectrum of normalised Ct values with a global *Z*-score: red indicates high level of expression and green indicates low level of expression. Non-hierarchical clustering corresponding to 16 selected genes is shown. Two clusters of cells can be identified with on the left the nascent fraction of plasma cell progenitors showing induced plasma cell transcription factors expression in green linked to an IL-2 signature (*IL2RA* expression), concomitant with B cell factors repression in red. **d** Heterogeneity of CD25 (IL2RA) expression analysed by flow cytometry in the D4-CFSE^lo^ subset of cells. Cell division visualised by CFSE dilution at D4 is similar in IL-2 treated and non-treated cells (IL2 and No IL2, respectively). Among CFSE^lo^ cells, CD25 was upregulated in a subset of cells that were cultured in presence of IL-2 (CD25^hi^). **e** IL-2-primed cells, sorted at D4 based on CFSE dilution and CD25 expression from gates in **d**, were put back in culture to assess differentiation into plasma cells (CFSE^lo^CD38^hi^) monitored by flow cytometry at D6, in comparison with their non-sorted counterpart (Total IL2-primed) and the control cells cultured without IL-2 (Total No IL2). One experiment representative of 5 is shown. **f** Absolute numbers of DAPI negative cells (Live cells) and CFSE^lo^CD38^hi^ plasmablasts generated from 10^5^ CFSE^lo^CD25^lo^ cells (in green) or CFSE^lo^CD25^hi^ cells (in purple) sorted at D4 from gates in **d**, and put back in culture for 2 more days (mean ± s.e.m, *n* = 5). For experiments shown in **b** and **f**, *p < 0.05, **p < 0.01, ***p < 0.005, NS: No significant differences, two-tailed unpaired Student’s *t*-test

Both, human peripheral blood CD19^+^CD27^−^CD10^−^ (mainly naive B cells) and highly pure mature ABCB1 transporter-positive naive B cells selected based on their capacity to extrude the mitotracker green fluorescent dye^27,28^, were differentiated into plasmablasts (CD20^lo^CD38^hi^) and plasma cells (CD138^+^) after 7 days of culture (Fig. 1a). This differentiation process combines B-cell activation initiated by BCR cross-linking, CpG synthetic oligonucleotides and CD40L, followed by a day-2 to day-4 (D2−D4) expansion of heterogeneous cell populations differing in their proliferative and differentiation capacities. At D4, cell division tracking using carboxyfluoroescein diacetate succinimidyl ester (CFSE) distinguished CFSE^hi^ from CFSE^lo^ activated B cell populations. The later population has previously been shown to differentiate into plasma cells when primed with IL-2 or IL-15, in the first 48 h of culture^21^. To address the ability of antigen-primed B cells to respond to transient IL-2 signal, we performed kinetic experiments. By D3 most of the cells were unresponsive to IL-2 while a short IL-2 stimulation at D2 conferred plasma cell differentiation ability to their progeny (Fig. 1b). However, among D4 CFSE^lo^ cells, only a subset gained the capacity to differentiate into plasma cells. Thus, to escape the averaging caused by B cell heterogeneity we implemented single-cell QRT-PCR to compare the D4 CFSE^lo^ cells that were primed with IL-2 with the non-primed ones. Unsupervised clustering analysis defined a minor subset of cells (30%) among the IL-2 primed cells characterised by the significant downregulation of B cell identity factors including *CD20*, *BACH2*, *BCL6*, *SPIB*, *IRF8* and *PAX5, p* < 10^−4^ (ANOVA) and the upregulation of plasma cell factors *PRDM1* and *XBP1s*, *p* < 10^−3^ (Fig. 1c, Supplementary Fig. 1). These committed cells had efficiently integrated IL-2-mediated STAT5 activation as demonstrated by the upregulation of the STAT5 target *IL2RA*, *p* < 0.005 (ANOVA). In agreement with the role of IL-2 in triggering ERK signalling that mediates plasma cell fate commitment^21^, the specific ERK-associated *MAPKAPK3* was upregulated in these cells, *p* < 10^−4^ (ANOVA). To link this signature with the ability of the cells to differentiate, the cell surface CD25 protein (IL2RA) was used to discriminate the committed cells at D4 within the CFSE^lo^ subset cultured in presence of IL-2 (Fig. 1d). CFSE^lo^CD25^hi^ cells and the CD25^lo^ counterpart were sorted and put back in culture for an additional 48 h prior differentiation monitoring by flow cytometry (Fig. 1e). On average, 76% ± 5% (*n* = 5) of the CFSE^lo^CD25^hi^ cells underwent differentiation whereas less than 10% of plasmablasts (9.8% ± 4%) emerged from the CFSE^lo^CD25^lo^. Importantly, this was not due to differences in survival as shown by the absolute number of plasmablasts generated in each condition (Fig. 1f). Thus the heterogeneity in B cell responses results from differences in early IL-2 signal integration.

### BACH2 silencing mimics IL-2-triggered differentiation

**Fig. 2:**
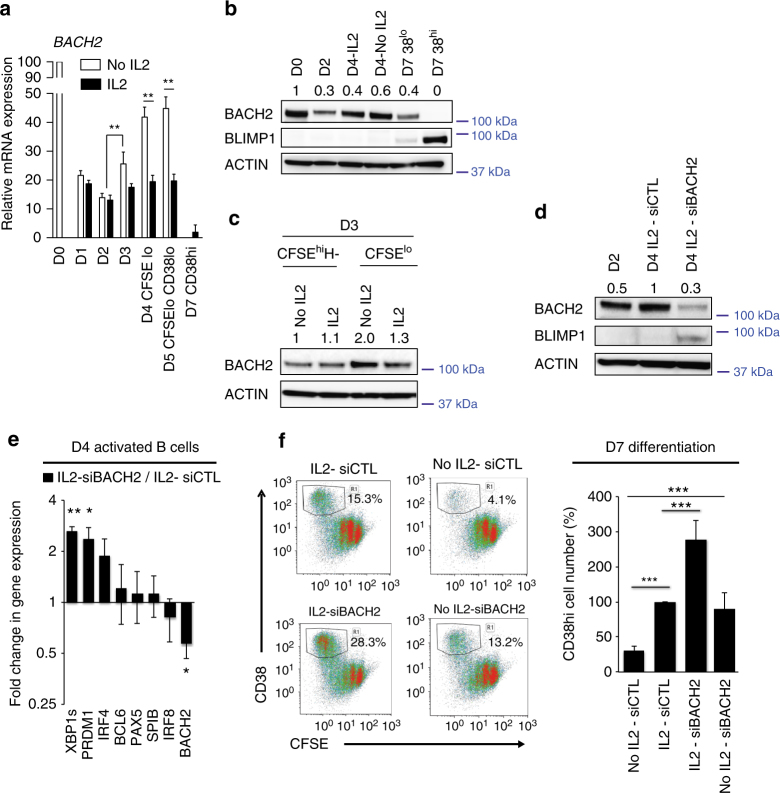
Transient inhibition of BACH2 expression induces plasma cell differentiation. **a** Time course analysis (D0−D7) of *BACH2* expression quantified by QRT-PCR and normalised to *HPRT1* during naive B cells differentiation in proliferative subsets (sorted CFSE^lo^ cells) and differentiated plasma cells (CD38^hi^). Naive B cells were stimulated with IL-2 during the activation phase (black bars) or not (No IL2, white bars). Expression in naive B cells (D0) was fixed to 100 and the experiments repeated on 6 different B cell donors. **b** Representative western blot for BACH2 and BLIMP1 protein levels at the indicated days of naive B cells culture. β-ACTIN was used as loading control. Values are the average fold changes in the expression levels of BACH2 normalised to β ACTIN for three independent experiments. **c** BACH2 protein levels in different subsets of D3-naive B cells stimulated or not with IL-2 for 24 h. Cells were sorted according to CFSE dilution and Hoechst (H) staining. CFSE^hi^ H^−^ corresponds to the non-proliferative subset. β-ACTIN served as loading control. This analysis was repeated two times. Values are the average fold changes in the expression levels of BACH2 normalised to β-ACTIN levels. **d** Protein expression levels of BACH2 and BLIMP1 analysed by immunoblotting, β-ACTIN was used as loading control. The efficiency of BACH2 inhibition with siRNA (siBACH2) was monitored at D4, 48 h after cell electroporation. Non-targeting siRNA (siCTL) was used as control. Values are the average fold changes in the expression levels of BACH2 normalised to β-ACTIN levels for four independent experiments. **e** Fold change in gene expression levels analysed by QRT-PCR in D4-IL-2-primed cells that were electroporated on D2 with siBACH2. Expression levels of three independent experiments were normalised to expression levels in the control cells (IL2-siCTL). **f** Flow cytometry analysis of CD38^hi^CFSE^lo^ plasmablasts generated at D7 following IL-2 priming (IL2) or in absence of IL-2 priming (No IL2) and electroporated with siBACH2 or siCTL at D2. The absolute numbers of plasmablasts were quantified by flow cytometry for seven independent experiments. Results were normalised to the IL2-siCTL condition arbitrary fixed to 100 for all the experiments. Values in **a**, **e**, **f** are shown as means ± s.e.m., *p < 0.05, **p < 0.01, ***p < 0.005, NS: No significant differences, Mann−Whitney test was used for **a** and **f**, two-tailed unpaired Student’s *t*-test for **e**

We previously showed that IL-2-induced ERK signalling leads primarily to a downregulation of *BACH2* ^21^. We thus monitored its expression in two culture conditions, with or without IL-2 added at D0 (Fig. 2a). Relative gene expression analysis showed a marked decrease of *BACH2* independently of IL-2 stimulation within the first 48 h of culture. Its expression increased significantly at D3 and in the D4 and D5 proliferative subsets in the absence of IL-2, *p* < 0.01 (Mann−Whitney test). In contrast IL-2 stimulation repressed *BACH2* expression between D2 and D4. This temporal regulation of *BACH2* was confirmed at the protein level (Fig. 2b, c) and raised the possibility that BACH2 may control the engagement of B cells in the differentiation path. To test this hypothesis, we used small interfering RNAs (siBACH2) to transiently inhibit *BACH2* expression at D2 through to D4. Knockdown efficiency was controlled by immunoblotting 2 days after electroporation (Fig. 2d, Supplementary Fig. 2a). Consistent with the known regulation of *PRDM1* by BACH2, an unimpeded BLIMP1 expression was observed in BACH2 deficient cells. At the mRNA level the expression of the plasma cell-specific transcription factors *PRDM1* and *XBP1s* was upregulated (*p* < 0.05, *t*-test) whereas the expression of the B cell-specific transcription factors *BCL6*, *PAX5*, *SPIB* and *IRF8* did not show any significant difference compared with the control IL-2-primed cells at D4, suggesting that BACH2 targets primarily the plasma cell transcriptional program (Fig. 2e). We next explored plasma cell differentiation by flow cytometry at D7 (Fig. 2f). BACH2 inhibition significantly increased the capacity of IL-2-primed cells to differentiate (*p* < 0.005, Mann−Whitney test). More importantly, *BACH2* inhibition was sufficient to drive differentiation in the absence of IL-2.

**Fig. 3:**
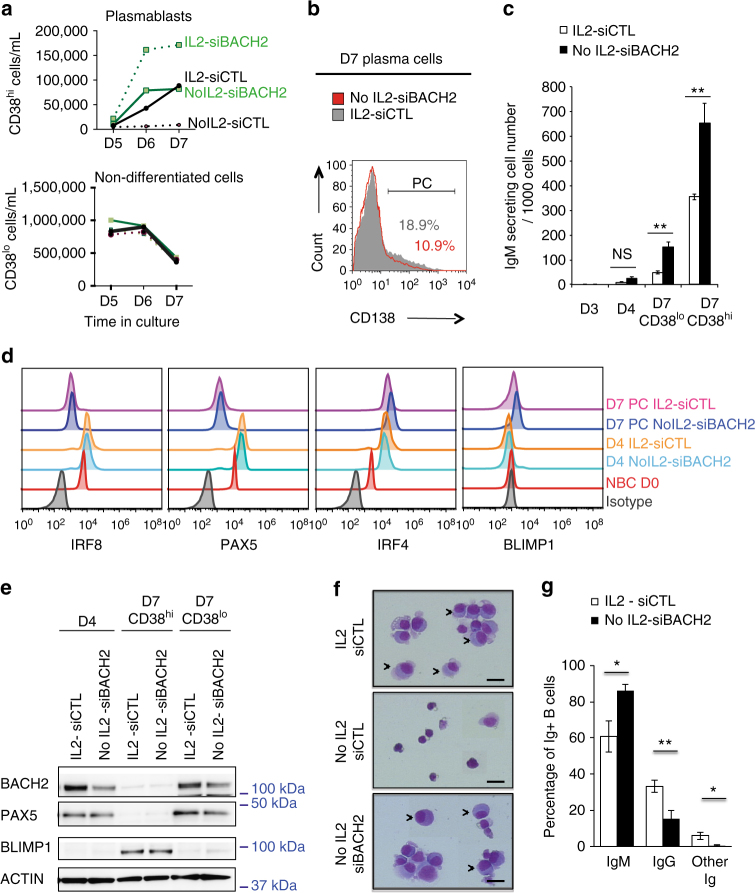
Differentiated B cells driven by siBACH2 exhibit a plasma cell identity. **a** Differentiation kinetics from D5 to D7 analysed by flow cytometry by the quantification of absolute cell number of plasmablasts CD38^hi^ (upper panel) and CD38^low^ (lower panel) generated from naive B cells that were electroporated with siBACH2 (green lines) or siCTL (black lines), primed or not with IL-2 (solid and dashed lines, respectively). A representative experiment from three independent experiments is shown. **b** Expression of the plasma cell marker CD138 analysed by flow cytometry at D7 within the differentiated CD38^hi^ compartment that were IL-2 primed (IL2-siCTL) or generated with BACH2 inhibition (No IL2-siBACH2). A representative experiment from four independent experiments is shown. **c** IgM secretion measured by ELISPOT at the indicated days and in sorted populations based on CD38 expression level at D7. A representative experiments from two independent experiments is shown. **d** Flowplots of B cell factors (IRF8, PAX5) and plasma cell transcription factors (IRF4, BLIMP1) expression by naive B cells (D0), D4-activated B cells and D7 sorted plasmablasts (PC, based on CD38^hi^ expression). Cells were primed with IL-2 when specified and electroporated at D2 with siBACH2 or the control siRNA. Corresponding isotypes were used as negative staining control. **e** Western blot analysis of BACH2, PAX5 and BLIMP1 expression by D4 and D7 sorted plasmablasts (CD38^hi^) and non-differentiated cells (CD38^lo^) that were primed or not with IL-2 and electroporated at D2 with siBACH2 or the control siRNA. **f** Cytospin and Giemsa staining of plasmablasts generated from BACH2 deficient naive B cells (No IL2-siBACH2) compared to controls. Representative cells are shown (×630 objective, scale bar 20 μm). They displayed features of plasmablasts (arrows), the nucleus is eccentric comparable to plasmablasts generated from naive B cells primed with IL-2 (IL2-siCTL). **g** Assessment of immunoglobulin class switching in CD38^hi^ cells at D7 generated from naive B cells deficient from BACH2 (No IL2-siBACH2) or cells that were primed with IL-2 (IL2-siCTL). Data are presented as percentage of positive cells for intracellular staining of IgM and IgG evaluated by flow cytometry. Means ± s.e.m. of four independent experiments. Significant differences are shown. For experiments shown in **c** and **g** *p < 0.05, **p < 0.01, NS: non-significant differences, two-tailed unpaired Student’s *t*-test

We then studied the kinetic of the differentiation. Only few plasmablasts emerged from the No IL2-siBACH2 condition at D5 (Fig. 3a). The maximum rate was obtained at D6 and maintained at D7. At that time plasma cell stage was reached for a small percentage of cells indicated by CD138 expression (9% ± 1.2; *n* = 5, Fig. 3b). Time course of immunoglobulin M (IgM) secretion (Fig. 3c), and of B cell and plasma cell transcription factors expression confirmed the kinetic of the differentiation (Fig. 3d, e). In coherence with the differentiation process triggered by IL-2 priming, differentiated cells induced by siBACH2 displayed low levels of PAX5 and IRF8 expression and high levels of BLIMP1 and IRF4 as compared to naive B cells. Moreover, differentiated cells induced by siBACH2 displayed morphological features of plasmablasts studied by Giemsa staining (Fig. 3f), but were mainly IgM (Fig. 3g). To demonstrate that BACH2 inhibition did not affect the proliferation capacity of activated B cells in vitro, we performed ModFit analysis on CFSE-stained B cells (Supplementary Fig. 2b). Together with 5-ethynyl-2′-deoxyuridine (EdU) and active caspase 3 labelling experiments we showed that BACH2 deficiency did not affect activated B cell expansion, survival, or proliferation (Supplementary Fig. 2c). Altogether these results suggest that the mechanism triggering plasma cell differentiation in BACH2-deficient B cells was independent of a proliferation or precocious differentiation effect. Furthermore, differentiation remained dependent of the differentiation stimuli anti-BCR, CpG and CD40L (Supplementary Fig. 2d). Finally to show the specificity of the BACH2 effect, we inhibited the expression of other crucial B cell identity factors including PAX5 and IRF8 identified previously downregulated by IL-2-mediated ERK activation^21^. Their transient repression did not confer plasma cell differentiation ability to activated B cells (Supplementary Fig. 2e). Taken altogether our data confirm the key role played by BACH2 in blocking the plasma cell differentiation program.

### BACH2 is a key effector of IL-2/ERK signalling

**Fig. 4:**
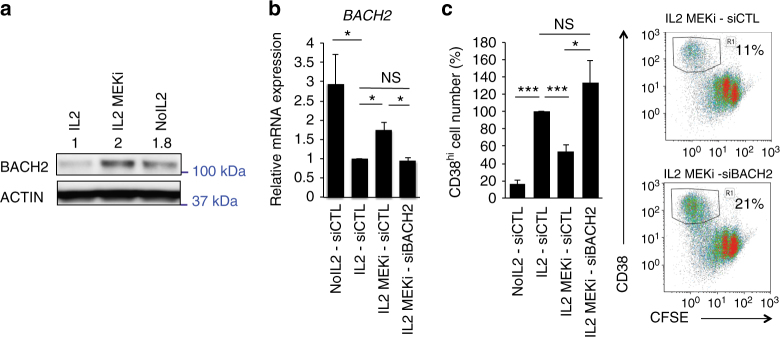
BACH2 mediates the effect of IL-2/ERK signalling on plasma cell differentiation. **a** BACH2 protein levels in D4-activated B cells primed in the absence (No IL2) or in the presence of IL-2 (IL2) and MEK inhibitor (MEKi) added on D2. β-ACTIN was used as loading control. Values are the average fold changes in the expression levels of BACH2 for three experiments. **b** Gene expression levels of *BACH2* in D4-activated naive B cells that were electroporated on D2 with siRNA and stimulated with or without IL-2 (IL2, No IL2, respectively) and treated with MEKi. Expression levels analysed by QRT-PCR were normalised to expression level in the IL-2 primed control cells from three independent experiments. **c** Plasma cell differentiation was monitored by flow cytometry at D7. CD38^hi^CFSE^lo^ cells were quantified for three independent experiments. Results were normalised to the IL2- siCTL condition arbitrary fixed to 100 for all the experiments. Values shown in **b** and **c** are means ± s.e.m., *p < 0.05, **p < 0.01, ***p < 0.005, NS: No significant differences, two-tailed unpaired Student’s *t*-test

We then wanted to determine whether BACH2 mediates the effect of IL-2/ERK signalling on plasma cell differentiation. To this end, we used MEK inhibitor (MEKi) to temporally and partially inhibit ERK activity at D2 in IL-2-primed cells, at a concentration that did not interfere with cellular proliferation but inhibited their ability to differentiate many divisions later^21^. We first validated that BACH2 expression, repressed by IL-2, was restored by ERK inhibition at the protein level (Fig. 4a). Then, D2 cells were transfected with siBACH2, treated with MEKi and subsequently stimulated with IL-2. At D4, *BACH2* expression levels were assessed. Partial inhibition of ERK signalling significantly restored *BACH2* expression in IL-2-primed cells, *p* < 0.05 (*t*-test) and siBACH2 reduced *BACH2* expression to a level similar to that in IL-2-primed cells (Fig. 4b). Strikingly, the ability of MEKi-treated cells to differentiate into plasmablasts was significantly rescued with the siBACH2, *p* < 0.05 (*t*-test, Fig. 4c). These findings indicate that *BACH2* is a key downstream target of IL-2 signalling.

### BACH2 controls IL-2-driven transcriptional programs

**Fig. 5:**
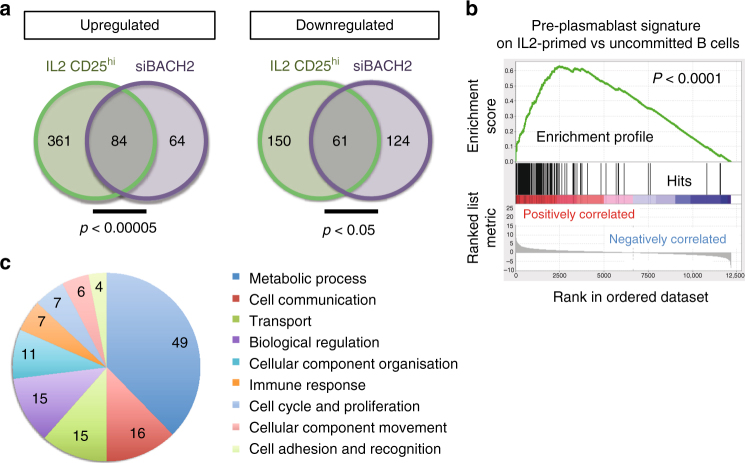
siBACH2-committed cells share an extensive transcriptional program with IL-2 primed cells. **a** Expression profiles of committed cells through IL-2 priming (IL2 CD25^hi^, sorted at D4 based on CFSE^lo^ and CD25^hi^ expression, in green) or through BACH2 transient inhibition (siBACH2 CFSE^lo^ cells sorted at D4, in purple). They were determined by RNA sequencing (*n* = 3 per condition) and compared to uncommitted cells (CFSE^lo^ cells that were cultured in absence of IL-2). A list of 445 and 211 genes that were, respectively, upregulated or downregulated after IL-2 priming were compared with the 148 and 185 genes that were, respectively, over- or under-expressed in siBACH2 compared with control cells (with a FC > 1.4 and p.adj < 0.05, Wald test) to generate Venn diagrams. The numbers of genes that are in common in both lists are indicated at the intersection between two circles. Hypergeometric distribution was used to calculate the *p*-value according to GSEA. **b** Gene set enrichment plot for genes highly expressed in preplasmablasts in the comparison between D4 IL-2-committed B cells and uncommitted B cells. **c** Functional analysis of upregulated genes in the BACH2 signature with the PANTHER classification system. The numbers of genes in each functional category are sown

To characterize the transcriptional program involved in the commitment towards plasma cell, we compared the RNA-seq gene expression profiles of D4 IL-2-primed cells (the CFSE^lo^CD25^hi^ cells, Fig. 1d) to the D4 CFSE^lo^ siBACH2 cells. As control we took the D4 CFSE^lo^ cells obtained in absence of IL-2. We established a list of 656 genes that were differentially expressed between the IL-2-committed cells and controls (445 genes upregulated and 211 genes downregulated, FC > 1.4 and Benjamini-Hochberg adjusted *p*-value (p.adj) < 0.05, Wald test), and a list of 333 genes that were differentially expressed between siBACH2-committed cells and controls (148 genes upregulated and 185 genes downregulated, FC > 1.4 and p.adj < 0.05, Wald test). Both lists were then compared and genes in common represented in the Venn diagrams (Fig. 5a, Supplementary Fig. 3 and Supplementary Data 1). We observed significant overlaps between IL-2 and BACH2 signatures confirming the major contribution of BACH2 in IL-2-triggered plasma cell fate commitment (*p* < 5.10^−5^ for upregulated genes and *p* < 0.05 for downregulated genes, hypergeometric distribution). The IL-2 signature was highly enriched in transcripts induced in pre-plasmablasts^29^ demonstrating that IL-2 signalling confers broad changes in gene expression associated with plasma cell differentiation (Fig. 5b). Interestingly, the strongest siBACH2 effect in committed cells was the upregulation of metabolic genes followed by genes devoted to cellular processes such as cell communication, transport, cell cycle and proliferation, cellular component organisation and immune response (Fig. 5c).

### Identification of BACH2 target genes

**Fig. 6:**
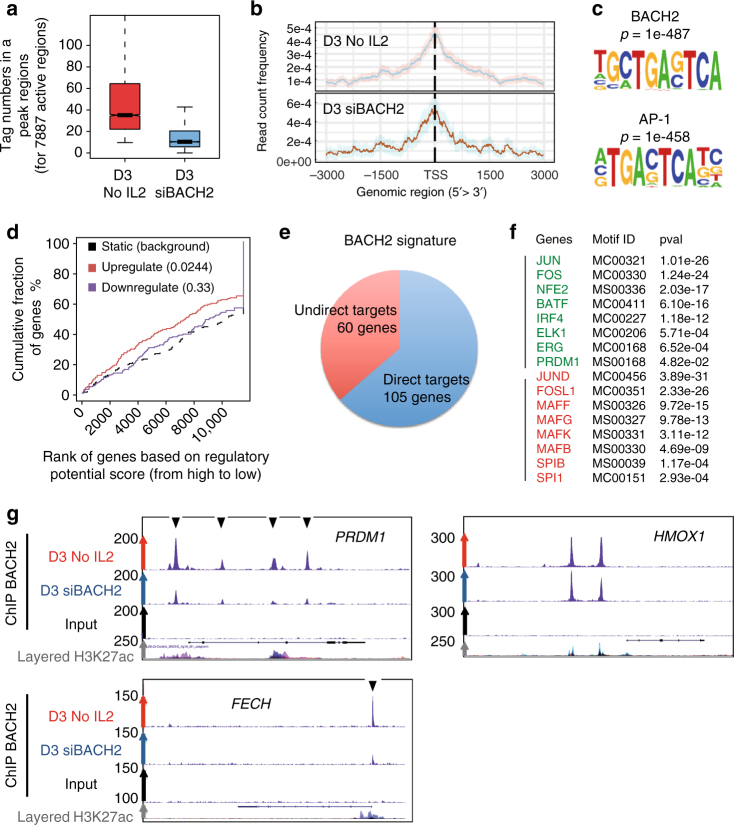
ChIP-seq integrated with transcriptomic data infers direct targets of BACH2. **a** Boxplots comparing peak size in 7887 active regions bound by BACH2 at D3 in activated naive B cells cultured in absence of IL-2 (No IL2). Reduced numbers of reads per regions were observed in siBACH2 condition compared to WT condition. Boxed area represents the centre 2 quartiles (notched-lines = median). Whiskers show the top and bottom quartiles without outliers. **b** Tag distribution (using bigWIG metrics) across transcription start sites (TSS). **c** Known motif enrichment in the ChIP-seq (D3 activated B cells, No IL2) using HOMER. The AP-1 consensus motif is embedded within the BACH2 motif. **d** BETA-plus analysis highlights activating/repressive function prediction of BACH2 in activating B cells. The red and blue lines represent the upregulated and downregulated genes, respectively. The dashed line indicates the non-differentially expressed genes as background; *p*-values represent the significance of the UP or DOWN group established by the Kolmogorov−Smirnov test. **e** Prediction of direct or indirect targets of BACH2. BETA-plus analysis was performed with BACH2 ChIP-seq data from D3 activated B cells and correlated with upregulated gene induced by siBACH2 in D4 CFSE^lo^ cells (p.adj < 0,05, Wald test). BETA package predicts direct regulation of 105 genes by BACH2, and 60 indirect targets. **f** Motif analysis in upregulated gene regions using basic setting of BETA software. Specific enrichment for transcription factor promoting plasma cell differentiation (green), or repressing this process (red) is shown. **g** UCSC genome browser visualisation of ChIP-seq data generated at D3 in activated naive B cells. Arrows represent ChIP-seq peaks of BACH2 binding that is decreased in the siBACH2 condition. Enforced BACH2 repression affects BACH2 bindings in *PRDM1* and *FECH* regulatory regions identified by H3K27Ac layer from ENCODE, but does not affect BACH2 binding in *HMOX1*

To infer the direct targets of BACH2, we mapped genome-wide BACH2 binding sites using ChIP-seq. To this end, we used activated B cells after 3 days of culture without IL-2; at that time BACH2 expression was increased (Fig. 2a, c). We took siBACH2-cells as control. After normalisation, 7883 regulatory regions were obtained compared with 1439 for the control-siBACH2 (Fig. 6a). We observed extensive binding at intergenic and intronic regions (Supplementary Fig. 4), while 20% of regulatory regions were located in gene promoters and enriched at transcription start sites (TSS, Fig. 6b). Motif enrichment analysis identified BACH2 consensus motif that embeds AP-1 binding sites (Fig. 6c). We then performed BETA analysis to integrate BACH2 bindings with differentially expressed genes identified above in the BACH2 repression condition. A repressive function for BACH2 was predicted, *p* < 0.05 (Kolmogorov−Smirnov test, Fig. 6d). Hereafter we focused our analysis on the 165 upregulated genes in the siBACH2 condition (FC > 1, p.adj < 0.05, Wald test), 105 genes were predicted to be direct targets of BACH2 (Fig. 6e, Supplementary Data 2). Motif analysis of these genes demonstrated enrichment of binding sites for both B and plasma cell transcription factors (Fig. 6f). ChIP-seq data confirmed BACH2 binding to *PRDM1* locus and inferred new targets linked to the commitment. Of particular interest was the ferrochelatase gene *FECH* required for haeme synthesis (Fig. 6g). Haeme homoeostasis plays a key role in plasma cell fate determination and CSR and high concentration of haeme inhibits BACH2 function^30^. Interestingly in T cells BACH2 was found to bind the haeme oxigenase gene *HMOX1* that in contrast to *FECH* is involved in haeme degradation^31^. Here we confirmed the binding of BACH2 in *HMOX1*; however, the binding was not affected in the siBACH2-cells, in line with our transcriptomic data showing no induction of *HMOX1* transcripts in the siBACH2 condition (Fig. 6g). Thus, FECH upregulation may be involved in haeme accumulation inhibiting BACH2 function in siBACH2 cells, a regulatory loop that may explain why a small difference in BACH2 expression level may tilt the balance in favour of plasma cell differentiation.

Three members of the dual-specific phosphatases (DUSP) family were identified as targets of BACH2 and found upregulated in the committed signature: *DUSP4*, *DUSP5* and *DUSP16* suggesting that the ERK pathway was ultimately under the control of inhibitory molecules in both commitment conditions. Indeed, *Dusp5* is required for murine plasma cell differentiation, inhibiting BCR-mediated ERK activation^25^.

Four transcription factors commonly upregulated in committed cells were bound by BACH2: *ID2*, *TOX2*, *PIR* and *ATF5*. ATF5 has a well-established pro-survival activity, regulating *MCL1* expression which is essential for GC formation^32^. Members of the *BCL2* family were also part of the BACH2 signature: the anti-apoptotic *BCL2L1* (BCLX_L_) previously described upregulated in GC B cells^32^, and the pro-apoptotic member *BCL2L15* whose expression was downregulated, revealing BACH2 contribution to a balance of the apoptotic signalling pathway. Other genes critical for GC homoeostasis were part of the BACH2 program such as *S1PR2* involved in GC B cell clustering and survival^33^.

### ELK1 controls *BACH2* expression

**Fig. 7:**
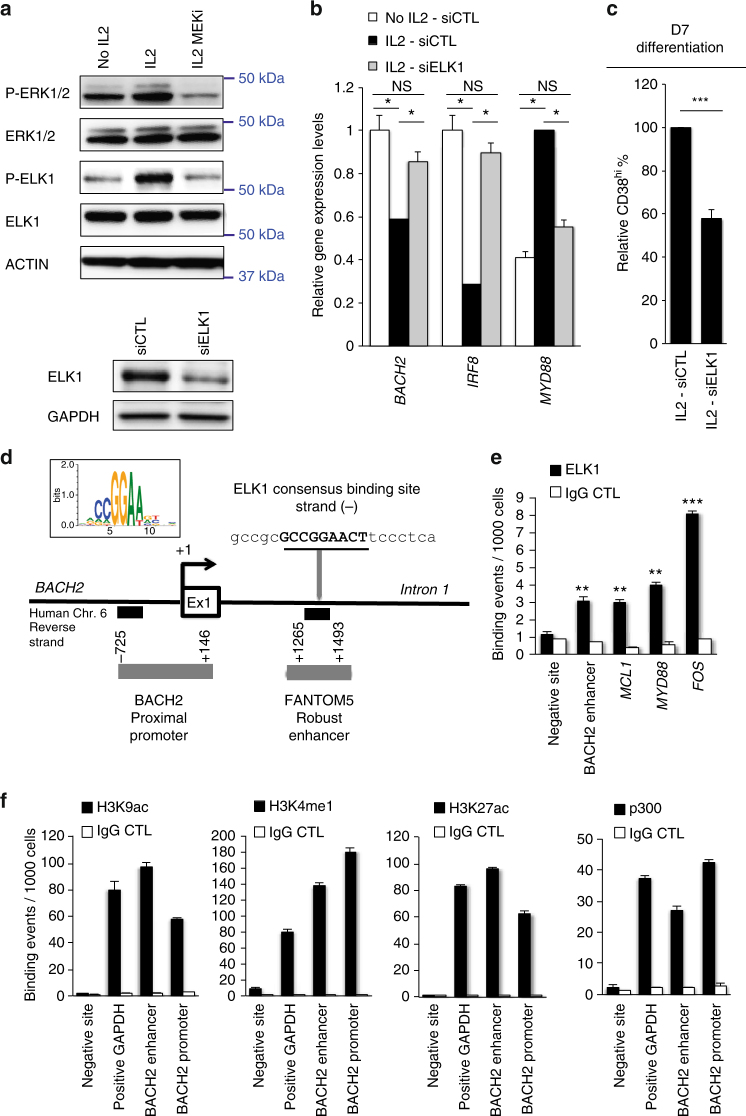
ELK1 is a mediator of IL-2 signalling and binds within *BACH2* super-enhancer. **a** Upper panel: MAP kinase activity analysed by phospho-specific immunoblotting in D2-activated B cells, starved and stimulated or not for 5 min with IL-2 and MEK inhibitor (MEKi) or DMSO; IL-2 signal triggers ERK and ELK1 phosphorylation (P-ERK1/2, P-ELK1) which is blocked with MEKi treatment. β-ACTIN was used as loading control. One representative of two experiments is shown. Lower panel: Protein expression levels of total ELK1 analysed by immunoblotting 2 days after siELK1 electroporation. GAPDH was used as loading control. **b** Effect of ELK1 inhibition on gene expression levels analysed by QRT-PCR in D3 B cells primed (IL2) or not (No IL2) with IL-2 for 24 h and electroporated at D1 with siELK1 or siCTL. *BACH2*, *IRF8* and *MYD88* expression levels in CFSE^lo^-cell sorted compartments were normalised to expression levels in IL-2 primed control-B cells. Means ± s.e.m. of three independent experiments. **c** Effect of siELK1 on the ability to differentiate into plasmablasts (CD38^hi^) in condition that favours plasma cell generation (IL-2 priming). Data from six independent experiments are presented relative to CD38^hi^ cell number generated by IL-2-primed and siCTL-electroporated cells at D1 (fixed to 100%). Means ± s.e.m. **d** Human *BACH2* locus including the position of the ELK1 consensus binding site (MATINSPECTOR prediction) in intron 1, within an enhancer region (FANTOM5 prediction) and the positions detected by QPCR for the ChIP assays within the proximal promoter and intron 1 identified by dark boxes. Numbers indicate positions relative to the TSS (+1). Ex1 means Exon 1. **e** ChIP assay of the in vivo binding of ELK1 in IL-2-primed D3 B cells. DNA immunoprecipitated by total ELK1 antibody or immunoglobulin G (IgG CTL) was amplified by real-time PCR using primers flanking the putative ELK1 binding site in *BACH2* intron 1 (predicted BACH2 enhancer position), known ELK1 binding regions in *MCL1*, *MYD88* and *FOS* genes, and negative control primers as a reference (negative site). Means ± s.e.m. of four independent experiments. **f** ChIP analysis of histone modifications and p300 binding in IL-2-primed D3 B cells. Immunoprecipitated DNA was amplified by QPCR using primers in the *BACH2* proximal promoter (BACH2 promoter) and predicted enhancer (BACH2 enhancer). Silent locus (negative site) and *GAPDH* specific positive control primers were used. Data are representative of two experiments (average and standard deviation of binding events per 1000 cells) with IgG control ChIP values (IgG CTL) shown separately. For experiments shown in **b**, **c**, **e**, *p < 0.05 **p < 0.01 ***p < 0.005, two-tailed unpaired Student’s *t*-test

To understand the mechanism by which IL-2 regulates *BACH2* expression, we searched for factors regulated by the ERK pathway and whose inhibition restores *BACH2* expression. Yasuda et al.^23^ provided evidences in mouse models for the control of Blimp1 expression by ERK/ELK1 signalling pathway. To test whether ELK1 is involved in IL-2-triggered plasma cell differentiation, we first realized western blot analysis that demonstrated phosphorylation of ELK1 by IL-2 stimulation, in an ERK-dependent manner (Fig. 7a). Next, we implemented siRNA experiments against *ELK1* at D1 to inhibit its expression prior to IL-2 stimulation. Knockdown efficiency was verified 2 days after B-cell electroporation at the transcript and protein levels (Fig. 7a, Supplementary Fig. 5a). We analysed the effect of ELK1 deficiency in D3 CFSE^lo^ cells. A known target of ELK1, *MYD88* ^34^ was used as control and found significantly repressed in ELK1 deficient B cells, *p* < 0.05 (*t*-test, Fig. 7b). In contrast *BACH2* and *IRF8* expression levels were significantly increased recapitulating the effects observed with MEKi, suggesting that IL-2-mediated ELK1 phosphorylation can suppress the expression of *BACH2*. In accordance with this finding, we observed a marked reduction in plasmablast generation in the siELK1 condition at D7, independently from a proliferation effect reinforcing the ELK1/BACH2 axis (Fig. 7c, Supplementary Fig. 5b).

### ELK1 binds *BACH2* intron 1 within a region of open chromatin

In silico motif search for transcription factor binding sites identified an ELK1 binding motif in the first intron of *BACH2* with an exact match with matrix consensus used by MatInspector (Fig. 7d). This region is highly CpG rich and shows more than 70% local nucleotide sequence identity between mouse and human (Supplementary Fig. 6a). Moreover, the sequence surrounding ELK1 binding motif (hg19: 91004796–91006944) was predicted to be a robust enhancer by the FANTOM5 project^35^, located in a super-enhancer structure identified by H3K27ac loading^16,17^ (Supplementary Fig. 6b). This region was thus hereafter called *BACH2* enhancer. We demonstrated an in vivo binding of ELK1 to this putative enhancer by chromatin immunoprecipitation followed by QPCR (ChIP-QPCR) in IL2-primed B cells collected at D3 (Fig. 7e). We also assessed binding of ELK1 to loci previously described in the literature including *MCL1*, *MYD88* and *FOS* ^34^ as controls. To examine whether ELK1 binding occurred within an open chromatin region, H3K9ac, H3K4m1, H3K27m1 and H3K27ac were studied by ChIP-QPCR. All these histone marks were present in the proximal promoter region of *BACH2* and in the enhancer (Fig. 7f). Moreover we examined p300 binding by ChIP-QPCR since it is a highly accurate means for identifying enhancers and their associated activities^36^. In line with an enhancer region, we observed marked enrichment of p300 binding at D3 (Fig. 7f). Interestingly, p300 time course analysis during naive B-cells activation mimicked, to some extent, BACH2 mRNA variations (Supplementary Fig. 6c). We then wanted to know whether ELK1 DNA-binding was modulated by IL-2 stimulation. To this end we performed ChIP-QPCR analyses in D3 cells that were stimulated or not with IL-2 (Supplementary Fig. 6d). No significant differences were observed between these two conditions, in agreement with previous studies demonstrating that ELK1 can be bound in the absence of stimulus^37–40^. Thus we hypothesised that IL-2/ERK may modify the transcriptional activity of ELK1.

### Mapping of a functional *BACH2* enhancer in activated B cells

**Fig. 8:**
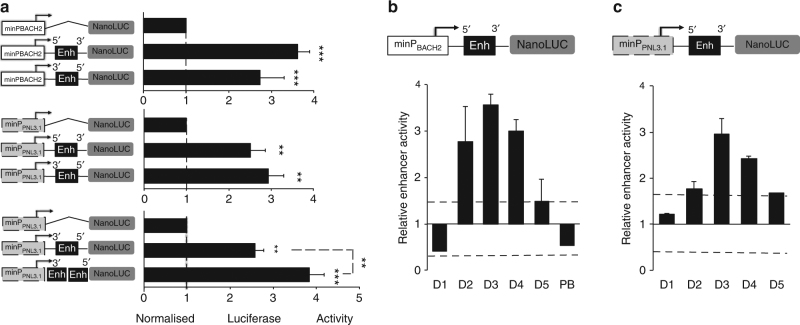
Enhancer activity of the 228 bp intronic sequence encompassing the ELK1 binding site. **a** Luciferase activity of primary activated naive B cells transfected with luciferase reporter plasmids for intron 1 enhancer (Enh) sequence (position +1265; +1493) ligated in either 5′-3′ or 3′-5′ orientations, made in conjunction with the proximal BACH2 promoter (minP_BACH2_, position −725; +146) or with the independent pNL3.1 minimal promoter (minP_PNL3.1_). Luciferase activity measured at D3, 24 h after electroporation, is presented relative to the promoter activity alone fixed to 1 (line1). Data are representative of three independent experiments (means ± s.e.m., **p < 0.01 ***p < 0.005, two-tailed unpaired Student’s *t*-test). **b** Temporal dynamic of the enhancer activity across naive B cells activation and differentiation assessed from D1 to D6 in sorted plasmablasts (PB). Activated B cells were electroporated with the reporter constructs carrying the enhancer sequence in conjunction with the *BACH2* promoter **b** or an independent minimal promoter **c**. Luciferase activity was measured 24 h later. Enhancer activity is presented relative to promoter activity alone arbitrary fixed to 1. Data are means ± s.e.m. from 6 (in **b**) and 3 (in **c**) independent experiments. Statistically significant cutoff values (dotted lines) were obtained by adding two standard deviations to the mean value obtained for the promoter activity

We decided to detail the functional DNA regulatory sequence by luciferase reporter assay in the context of primary human naive B cell activation. The *BACH2* proximal promoter^41^ (minP_BACH2,_ −725; +146) ligated upstream from the nano-luciferase coding sequence (NanoLuc) demonstrated a 4.2 ± 0.2 (*n* = 15) fold more transcriptional activity than the promoterless NanoLuc vector (pNL1.1), whatever the time point of B-cell electroporation (from D0 to D3). As minP_BACH2_ did not respond substantially to the B-cell activation cocktail, it was used to test the transcriptional activity of ELK1. The enhancer region (+1265; +1493) was inserted in the native and reverse orientation downstream from the minP_BACH2._ The addition of this sequence resulted in a threefold to fourfold increase in luciferase activity measured at D3 (Fig. 8a, upper panel). A similar effect was observed when the *BACH2* intronic sequence was ligated downstream from an independent promoter (minP_PNL3.1_) (Fig. 8a, middle panel). Finally, an additive effect on transcriptional activity was observed when 2 copies of the sequence were inserted in the plasmid (Fig. 8a, lower panel) conform to a functional enhancer sequence. We then studied the dynamic of its activity during naive B-cell activation and differentiation triggered by IL-2. For each time point analysed, the cells were electroporated 24 h before with the enhancer sequence in conjunction with minP_BACH2_ (Fig. 8b) or with minP_PNL3.1_ (Fig. 8c). No activity was observed at D1 or in sorted D6 plasmablasts. Time course analysis however revealed the dynamic activation of the enhancer starting from D2, up to D4, with a peak at D3. Interestingly the kinetic activity of the enhancer mimicked the upregulation of *BACH2* mRNA observed between D2 and D4, which was more pronounced in the absence of IL-2 (Fig. 2a). These results suggested that this functional sequence might control *BACH2* expression during the critical IL-2-mediated molecular switch responsible for early B cell engagement towards plasma cell differentiation.

### ELK1 and cell division regulate BACH2 enhancer activity

**Fig. 9:**
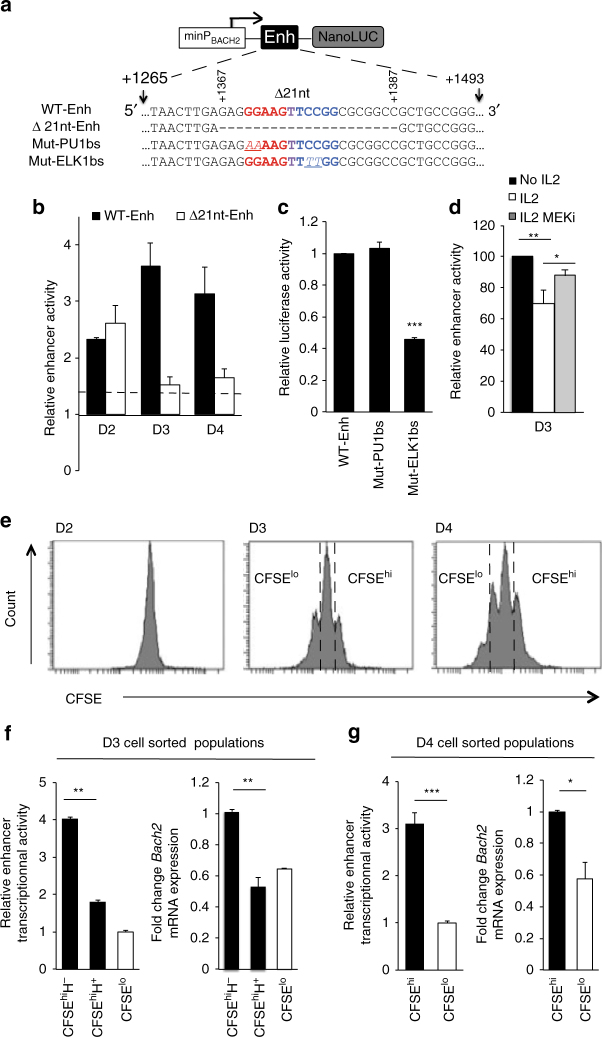
Role of the ELK1 binding motif in *BACH2* enhancer. **a** ELK1 binding motif (core motif in blue) partially overlaps PU.1 predicted binding motif (core motif in red) within the enhancer of *BACH2* intron 1. Positions refer to +1 transcription start site. Deletion of 21 nucleotides from the enhancer (Δ21 nt-Enh) or point mutations in the core motifs of PU1 binding site (Mut-PU1bs) or ELK1 binding site (Mut-ELK1bs) are underlined. **b** Luciferase activity of activated naive B cells electroporated with the wild-type enhancer (WT-Enh) or the enhancer deleted of 21 nucleotides (Δ21 nt-Enh), at different time points (D1, D2 and D3) and cultured for 24 h. Luciferase activity is presented relative to the cells electroporated with the BACH2 promoter (minP_BACH2_) alone (activity fixed to 1 for the different time points). Statistically significant cutoff value (dotted line) was obtained by adding 2 standard deviations to the mean value obtained for the promoter activity alone. **c** Luciferase activity measured at D3, 24 h after electroporation of reporter construct that carries the wild-type enhancer sequence (WT-Enh) and the derivative plasmids with mutations in PU1 core motif (Mut-PU1bs) or ELK1 core motif (Mut-ELK1bs). Luciferase activity is presented relative to the activity of the WT-Enh. (*n* = 3, mean ± s.e.m., ***p < 0.001, two-tailed unpaired Student’s *t*-test). **d** Effect of IL-2 stimulation on the enhancer activity. Activated B cells were electroporated on D2 with the WT enhancer reporter construct and then stimulated (IL2) or not (No IL2) with IL-2 for 24 h in presence of MEK inhibitor (MEKi) or DMSO. Enhancer activity (fixed to 100 for the No IL2 condition) was determined relative to luciferase activity of the BACH2 promoter alone. (*n* = 6, mean ± s.e.m., **p = 0.007, two-tailed unpaired Student’s *t*-test). **e** Kinetic of CFSE profiles. **f** Enhancer activity measured by luciferase assay and *BACH2* gene expression level at D3 in **f** and D4 in **g**, in cell-sorted populations based on CFSE dilution from gates in **e** (CFSE^hi/lo^), and cell cycle: Hoechst positive (H^+^) vs. Hoechst negative (H^−^). Data are representative of three independent experiments (means ± s.e.m., *p < 0.05, **p < 0.01, ***p < 0.005, two-tailed unpaired Student’s *t*-test)

We then tested the contribution of the ELK1 motif to the temporal activation of the enhancer. First we made serial deletions from the enhancer and measured the transcriptional activities at D3. Deletion of the ELK1 binding motif (positions +1367 to +1387, Supplementary Fig. 7) resulted in diminished luciferase activity almost equal to that of the minP_BACH2_, in line with a positive role of ELK1 motif. Then, reporter constructs carrying the full-length enhancer (referred as WT-Enh) or the enhancer sequence deleted of 21 nucleotide (nt) corresponding to the ELK1 motif (referred as Δ21nt-Enh) (Fig. 9a) were transfected in activated B cells at different time points of the culture and luciferase activities were measured 24 h later (Fig. 9b). We confirmed the positive role of the 21 nt sequence at D3 and D4 as its deletion resulted in diminished activity almost equal to that of the minP_BACH2_. In contrast, analysis performed in D2-activated B cells showed lower enhancer activity compared to that performed at later time points, and the 21 nt deletion did not demonstrate any substantial effect on the luciferase activity (Fig. 9b). These data indicated that different regulatory elements contributed to the enhancer function at different time point of the activation process.

In addition to the predicted binding motif for ELK1 in the 21 nt sequence, we identified a putative motif for PU.1 in the opposite strand of DNA, partially overlapping the ELK1 motif (Fig. 9a). To demonstrate the direct binding of ELK1 to its putative binding site and to exclude the possibility that PU.1 was responsible for the enhancer activity at D3, we performed point mutations in one of the two motifs. Inactive PU.1 motif (Mut-PU1bs) did not affect luciferase activity while inactive ELK1 motif (Mut-ELK1bs) led to a marked decreased of luciferase activity (Fig. 9c). Thus ELK1 binding contributed to enhancer function.

Next we investigated the responsiveness of the enhancer to IL-2 signal at D3. In line with IL-2-induced *BACH2* repression, we were able to show that IL-2 counteracts the enhancer activity although these experiments were done in heterogeneous cell populations (Fig. 9d). By partially inhibiting the ERK signal in the presence of IL-2 we observed a significant rescue of enhancer activity (*p* < 0.05, *t*-test) suggesting that sustained ERK activity may be responsible for maintaining or switching ELK1 under its repressive form, although the possible involvement of other regulatory elements cannot formally be excluded. Thus we proposed that IL-2 stimulation is involved in the ELK1 switch from an activating to a repressive form following ERK activation above a threshold. A similar mechanism was described upon mitogenic stimulation by Yang et al.^42^.

**Fig. 10:**
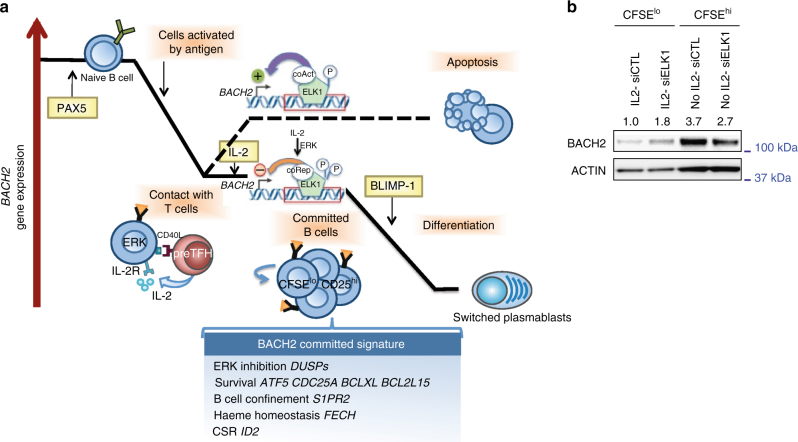
Proposed model of *BACH2* regulation during terminal B cell differentiation. **a** *BACH2* is highly expressed in naive B cells, under PAX5 control. Its expression decreases with antigen priming. In absence of T cell help, BACH2 acts as a key lock in repressing the plasma cell transcriptional program. *BACH2* expression is under the control of the transcriptional activity of an enhancer mediated by ELK-1 in association with co-activator (coAct) complex. Contact with cognate helper T cells and signals that sustained ERK signalling instruct a dynamic switch in ELK1 transcriptional activity that may recruit a corepressor (coRep) complex responsible for *BACH2* downregulation and plasma cell fate commitment. The IL-2-primed activated B cells that have proliferated (CFSE^lo^CD25^hi^) are committed for plasma cell differentiation. The differentiation program initiated by IL-2 revealed the major contribution of *BACH2* downregulation. RNA-seq and ChIP-seq revealed the broad program of molecular and cellular processes regulated by BACH2 at this early time point of B cell fate commitment. **b** BACH2 expression levels analysed by immunoblotting in subpopulations of D4-activated B cells sorted based on CFSE dilution. Naive B cells were primed or not with IL-2 and electroporated with siELK1 or a control siRNA (siCTL). β-ACTIN was used as loading control. Values are the average fold changes in the expression levels of BACH2 normalised to β-ACTIN levels for three independent experiments

ERK activity has been linked to B cell proliferation and some evidence suggests that B cells would integrate T cell derived signals, in addition to BCR signals, in a cell cycle-dependent manner^43^. Furthermore cell proliferation affects *BACH2* expression^22^. Thus, to explore whether proliferation can modulate *BACH2* expression and the enhancer activity, we profiled *BACH2* expression and enhancer activity at D3 and D4 in sorted populations based on CFSE dilution (Fig. 9e) and cell cycle (Hoechst staining). A marked difference in enhancer activity was observed in CFSE^lo^ compared to CFSE^hi^ cells at both time points (Fig. 9f, g). In line with enhancer contribution to *BACH2* expression, *BACH2* levels mimicked enhancer activities with higher enhancer activity and *BACH2* expression level being observed in CFSE^hi^ Hoechst-negative (G0/G1 phases) cells compared to Hoechst-positive (S and G2/M phases) cells (Fig. 9f). Collectively, these results characterize a *BACH2* enhancer under ELK1 control that may contribute to maintain *BACH2* expression, and when ERK activity becomes sustained with IL-2 stimulation and/or cell divisions, it may switch from an active to an inactive/repressive form, underlying the decline of *BACH2* expression and plasma cell commitment (Fig. 10a). Supporting this model, ELK1 contributed to the upregulation of BACH2 expression in the non-proliferative subset isolated at D4. In contrast, ELK1 repressed BACH2 expression in the CFSE^lo^ cells stimulated with IL-2 (Fig. 10b).

## Discussion

This study established a new link between early T cell help, intracellular signalling pathways, regulation of effector transcription factors expression and the B cell response through the characterisation of the IL2/ERK/ELK1/BACH2 axis which governs the B cell capacity to differentiate into plasma cell in a model system of primary human naive B cell culture. Strength in this model lies within the preliminary phase of activation as the full activation and proliferation of naive B cells in response to antigen-priming and T cell help (CD40L and cytokines) closely recapitulates the minimal initiation events observed during the first 4 days of an in vivo T cell-dependent response^44,45^. The sustained presence of exogenous anti-BCR during this phase is reminiscent of the readily accessible antigen presented by dentritic cells within the interfollicular zone, while soluble CD40L and IL-2 recreate the consecutive interactions undertaken by cognate B and T cells in vivo^46,47^. This T cell help activates B cells above a threshold required for eliciting plasma cell response, an effect mediated by BACH2 repression (Fig. 10a). This finding is in accordance with the inversed relationship observed in mice between the strength of T cell help received by B cells in the GC and *Bach2* expression levels^13^. This is also in line with the synergistic effect of IL-2 and IL-21 observed in our culture system confirming observations made by others^48^. IL-21 is presumed to be the most potent inducer of B cell terminal differentiation, an effect mediated by STAT3 activation and BCL6 repression^49,50^. Beside its differentiation effect and unlike IL-2, IL-21 enhanced cell proliferation of human activated B cells, which complicate the study of the underlying molecular mechanisms. We decided therefore to focus our study on the minimal IL-2-mediated ERK signalling triggering plasma cell differentiation through *BACH2* downregulation. We believe this IL-2/ERK axis could mimic in vivo situations when ERK is activated above a threshold potentially through different stimuli such as IL-4 or IL-15, or as a result of intense proliferation. In this context IL-21 that promotes proliferation may also contribute to the activation of this pathway. At least, a recent study demonstrated that antigen-primed murine B cells have a maximal 2-day window for acquisition of T cell help^51^. This finding is again in line with our study and the ability of human naive B cells to engage the plasma cell differentiation program in response to a very short duration of IL-2 stimulation within the first 2 days in culture (Fig. 2b). This is also in coherence with the short half-life of this cytokine^52^ and the transient secretion of IL-2 by the T cells^53,54^.

Defined differentiation stimuli in our model system contributed to dissect heterogeneity in B cell responses, which is an important challenge in investigating B cell physiology. Using single-cell QPCR we found that only a small fraction of cells were early committed to plasma cell differentiation. Our integrating transcriptomic and BACH2 chromatin binding data allowed the identification of a BACH2 gene-signature in these cells, what revealed a significant BACH2 contribution at the early stage of B-cell activation. Some genes of this signature are known for their role in both the GC biology and lymphomagenesis. *ATF5* for instance was found overexpressed in lymphoma and was recently associated with transformation to aggressive form of follicular lymphomas^55,56^. Several members of the BCL2 family were found regulated by BACH2 in this study. Oncogenic processes may corrupt the balance of the apoptotic-signalling pathway under BACH2 control leading to cell proliferation and tumour progression. In fact, cumulative evidences exist for a role of BACH2 in lymphomagenesis, including the description of chromosomal translocations and mutations involving *BACH2* in some lymphomas^57–59^.

Our study reveals a mechanism involved in the temporal regulation of *BACH2* expression that control-B cell fate destiny (Fig. 10a). *In vivo* BACH2 controls a specific time frame where AID expression/activity is fully efficient until *PRDM1* expression is induced^6^. By taking in account those elements it is highly probable that BACH2 expression is finely regulated to enable immunoglobulin affinity maturation and to avoid unwanted genome-wide damages. In this study, our IL-2/ERK/BACH2 pathway fits with such fine-tune regulation of *BACH2* expression. The enforced repression of BACH2 in recently activated B cells recapitulated the phenotypes reminiscent of *Bach2*-deficient B cells in mice: the unimpeded BLIMP1 induction, a higher frequency of differentiated cells and a defect of CSR^6^. The unimpeded *PRDM1* expression could explain the impaired CSR observed in our model system. However beyond this mechanism we identified *ID2* as a direct target of BACH2. ID2 inhibits E proteins such as E2A involved in *AICDA* (encoding AID) expression, thus regulating CSR^60,61^. Therefore our study suggests that BACH2 expression may sustain *AICDA* expression through the repression of *ID2*.

Another insight into the effector functions of BACH2 at this early time point of B cell fate decision was its implication in mitochondrial metabolism and haeme homoeostasis. Herein we provide the first evidence that BACH2 regulates *FECH* expression encoding a key enzyme required for haeme synthesis. We propose a regulatory loop initiated by BACH2 repression, triggering haeme synthesis and consequently completing BACH2 inhibition by impairing its function.

Our data have shown that small differences in the expression levels of *BACH2* at critical time point of B-cell activation have consequent effects on B cell fate. We characterised a novel *BACH2* enhancer whose activity was dynamically regulated along the differentiation process. ELK1 was identified as the mediator of this IL-2-induced mechanism, accessing and binding to this new enhancer. ELK1 is a member of the ETS family of transcription factors at the crossroads of mitogen-activated protein kinase (MAPK) signalling cascades^62^. Its phosphorylation at S383 has mainly been studied, meanwhile different phosphorylation states and patterns of ELK1 exist and may vary with the stimuli triggering ERK activation thus determining the transcriptional response^63^. During the critical D2−D4 time window of BACH2 regulation, the *BACH2* enhancer activity was under ELK1 positive control perhaps resulting from summed signal input integration (BCR, CD40L and TLR9 signalling). In contrast, IL-2 input seemed to repress both the transcriptional activity of the enhancer and *BACH2* expression in ERK-dependent manner. Thus we propose that IL-2 signalling may be involved in ELK1 activator-repressor switching following ERK activation above a threshold (Fig. 10a). A similar mechanism was described earlier by Yang et al.^42^ involving the temporal recruitment of corepressor complex depending on the dynamic phosphorylation status of ELK1. Collectively, our data suggest that this enhancer is important for *BACH2* regulation in activated B cells and may provide a mechanism behind the Elk1-mediated upregulation of Prdm1 described earlier in a mouse model^23^.

In conclusion, our study has demonstrated a remarkably sensitive mechanism controlling the diversification and destiny of activated B cells. A short extrinsic input sustaining ERK activation initiates a molecular switch of ELK1 acting within the *BACH2* super-enhancer and fine-tuning *BACH2* expression. BACH2 regulates of a broad program of molecular and cellular processes that may have if disturbed potentially important implications for antibody response efficiency and in lymphomagenesis.

## Methods

### Primary B cell purification

Peripheral blood mononuclear cells from healthy volunteers were obtained from the Etablissement Français du Sang (Rennes, France) after Ficoll density centrifugation (Sigma-Aldrich). Volunteers were recruited under French Ministry of Higher Education and Research approval (AC-2014-2315) with written informed consent according to the Declaration of Helsinki. Human naive B cells were purified by negative selection using magnetic cell separation (Naive B Cell Isolation Kit II; cat no. 130-091-150 from Miltenyi Biotec) with anti-CD10 (0.5 μL/M of cells, cat no. 130-093-451 from Miltenyi Biotec) added in the antibody cocktail, using the AutoMACS deplete-sensitive program. The purity of the isolated CD19^+^ CD27^−^ naive B cell population was routinely >99%. For experiments involving CD19^+^CD27^−^MTG^−^ naive B cells, the CD19^+^ CD27^−^ population was stained with 100 nM MitoTracker Green FM (ThermoFisher) for 25 min at 37° and chased for 3 h (RPMI, SVF10%, 37°) before cell sorting populations on the BD FACSAria cell sorter (BD Biosciences).

### Cell culture and cell sorting

Cell culture conditions, antibodies and flow cytometry procedures are as described in ref. ^21^ with a few modifications. All cultures were performed in complete medium consisting of RPMI 1640 (Invitrogen) supplemented with 10% FCS (Biowest) and antibiotics (Invitrogen). Apoptosis and proliferation were analysed using a PE-conjugated anti-active caspase-3 apoptosis kit (cat no. 550914 from BD Biosciences) and Click-IT Plus EdU Alexa Fluor 647 Flow cytometry assay kit (cat no. C10634 from ThermoFisher), respectively, according to the manufacturer’s instructions. CFSE labelling of naive B cells was performed with 1 μM CFSE (Invitrogen) in serum free medium at 37 °C for 10 min and washed in complete medium to follow cellular divisions (ModFIT analysis—VSH), and enable cell sorting of CFSE^hi/lo^ populations. Purified naive B cells were cultured at 7.5 × 10^5^ cells/ml in 24-well plates and stimulated during 4 days with 2.6 μg/ml F(ab′)_2_ fragment goat anti-human IgA + IgG + IgM (H + L) (Jackson ImmunoResearch Laboratories), 100 ng/ml recombinant human soluble CD40L (NCI), 1.0 mg/ml CpG oligodeoxynucleotide 2006 (Cayla Invivogen), and 50 U/ml recombinant IL-2 (SARL Pharmaxie). ERK1/2 activation was inhibited with MEKi 0.5 μM (PD184161, Calbiochem). Day 4-activated B cells were washed and cultured at 4 × 10^5^ cells/ml for up to 3 days with 50 U/ml IL-2, 12.5 ng/ml IL-10, and 5 ng/ml IL-4 (R&D Systems). All Abs used for flow cytometry analysis are listed in Supplementary Table 1 For QRT-PCR analyses and luciferase reporter assays, CFSE-stained B cells were collected at the required time point and cell sorted into CFSE^hi/lo^ populations using FACsARIA (BD Biosciences). All flow cytometry gating strategies are shown in Supplementary Fig. 8.

To identify the minimal time and duration for IL-2 stimulation during the activation phase, anti-IL-2 neutralising Ab or anti-IL-2 receptor *α*, *β*, *γ* Abs with their isotype controls (all from R&D Systems) were used at 10 μg/mL in the medium.

For IgM secretion assessed by ELISA, D6-differentiated cells were sorted as CD20^low^CD38^hi^ and reseeded with IL-2, IL-4 and IL-10 at 5×10^5^ cells/mL for 24 h. IgM antibodies were detected using AffiniPure Goat Anti-Human IgA + IgG + IgM (H + L) (Jackson Immuno-Reseach Laboratories) coated on 96-well plates, followed by secondary peroxydase-conjuged goat anti-human IgM (Jackson Immuno-Reseach Laboratories). Purified Chrompure Human IgM (Jackson Immuno-Reseach Laboratories) was used as standard. Plates were developed using TMB Substrate Reagent Set (BD Biosciences). For ELISPOT, D6-sorted plasmablasts (100 and 500 cells, in triplicate) were grown for 1 h in 96-well plates with anti-IgM coated membranes (SIGMA). Secreting cells were detected with secondary anti-human IgM-peroxydase antibody (SIGMA). For both experiments, D6-non-differentiated B cells were used as negative control.

Cytologic analysis of differentiated cells was performed by preparing 10^5^ D6-cells for microscopy using cytospin followed by May-Grumwald-Giemsa staining.

### QRT-PCR analysis

RNA was extracted using RNeasy microkit (cat no. 74004 from Qiagen) and reversed transcribed into cDNA with Superscript II (Invitrogen). QRT-PCR was performed using the TaqMan Gene Expression Master Mix and run on the Step One Plus Real-time PCR System from Applied Biosystems. All TaqMan primers (Applied Biosystems) used in this study are listed in Supplementary Table 2. Gene expression levels were quantified using *HPRT1* as endogenous control. The 2 exp(−ΔΔCt) method was used to determine the relative expression of each gene.

For single-cell QRT-PCR experiments, gene expression levels for the 16 selected Taqman assays within single-cell cDNA were measured with qPCR on 96.96 Dynamic Array IFC using the Fluidigm BioMark HD system. A total of 285 single cells from C1 captures were profiled using Dynamics Array IFC. Hierarchical clustering was performed on R version 3.2.4 with ComplexHeatmap (R package version 1.6.0), fluidigmSC (Fluidigm SINGuLAR Analysis Toolset, R package version 3.5.2), dendextend, amap (R package version 0.8–14) packages. We used a conservative Ct of 24 as LOD based on published guidelines^64^_._ Gene expression was defined on a log2 scale as: log2 expression = LOD-Ct. Samples were clustered by the Euclidean distance, and genes were clustered by the pearson distance. Heatmap was generated with the ‘global_z_score’ (normalisation by the expression value with the global mean and the global standard deviation).

### Western blotting and DNA-binding ELISA

All antibodies used for western blotting are listed in Supplementary Table 3. 0.8–1 × 10^6^ primary naive B cells were recovered for protein extraction, which was performed using RIPA lysis buffer (Pierce) followed by cycles of sonication performed using the Biorupter Sonicator (Diagenode). Protein concentration was analysed using the BCA Protein Assay kit (cat no. 23225 from Pierce) and read by the Model 680 Microplate reader (Bio-Rad). Proteins were separated by the NuPAGE SDS-PAGE Gel system (Thermo Scientific) and transferred onto Immobilon-P PVDF membranes (Sigma-Aldrich) according to standard procedure. Detections were performed with HRP-conjugated secondary Ab (Bio-Rad) and enhanced chemiluminescent (ECL Plus) reagent (Amersham) using the G:BOX Chemi imaging system (Syngene). GAPDH or β-ACTIN on the same membrane served as loading control. Uncropped original scans of immunoblots are provided in Supplementary Fig. 9.

### Sequence alignment and regulatory elements identification

MatInspector^65^, TFBIND^66^, TFSEARCH (http://cbrc3.cbrc.jp/papia/howtouse/howtouse_tfsearch.html), PROMO^67^ and comparative genomics tool rVISTA^68^ were used for the identification of regulatory elements in human *BACH2* locus. BLAST program (NCBI) was used to search for alignment.

### ChIP-QPCR assay and analysis

ChIP-IT PBMC kit (cat no. 53042 from Active Motif) was used according to the manufacturer’s instructions. D3-activated naive B cells primed with IL-2 were fixed according to the protocol and lysed by sonication using the Epishear probe sonicator and the cooled sonication platform (Active Motif). Sonicated chromatin was controlled on gel prior to immunoprecipitation. Rabbit antibodies to ELK1 (ab32106), H3K9ac (ab4441), H3K27ac (ab4729), H3K4me1 (ab8895), p300 (ab10485) and IgG (ab37415) were from Abcam. Results were analysed with the ChIP-IT qPCR Analysis kit (cat no. 53029 from Active Motif) to calculate binding events detected per 1000 cells. Human Negative Control Primer Set1 and Human Positive Control Primer Set GAPDH^−^2 from the ChIP-IT qPCR Analysis kit were used, other primers are listed in Supplementary Table 4.

### siRNA experiments

All siRNAs (ON TARGET Plus, SMART Pool, Dharmacon) used in this study are listed in Supplementary Table 5. 10^6^ primary naive B cells per condition were recovered from culture for transfection using the Amaxa Cell Line Nucleofector Kit V (cat no. VCA-1003 from Lonza). Naive B cells were centrifuged at 1800 r.p.m. for 10 min at room temperature, re-suspended in transfection buffer and combined with 100 pmol of either target or control siRNA. Naive B cells were then electroporated using program O-17 of the Amaxa Nucleofector II Device (Lonza), re-suspended with pre-incubated media and cultured at 37 ^o^C for 24 or 48 h. Transfection was optimised using a labelled siRNA control (AF647). 75% of the electroporated cells were positive 24 h after electroporation with 80–90% cell viability. Knockdown mRNA efficiency was determined for each siRNA by QRT-PCR and western blot analysis.

### Luciferase reporter constructs

All oligonucleotides used in the construction of the *BACH2* luciferase reporter plasmids were designed using Primer3 and synthesised by Eurogentec. The list of cloning and sequencing primers is available in Supplementary Table 6. DNA insert sequences were amplified by PCR using Q5 High-Fidelity DNA polymerase (NEB) with primers containing restriction sites followed by PCR product purification (NucleoSpin Gel and PCR Clean-up, Machery-Nagel). Purified insert DNAs together with the appropriate expression vectors were then restriction digested using corresponding enzymes (CutSmart restriction endonucleases, NEB), purified and ligated together using T4 DNA ligase (Roche). The reporter vectors implemented for DNA insertion were the basic vector pNL1.1[*Nluc*] and minimal promoter vector pNL3.1[*Nluc*/minP] that both encoded the NanoLuc luciferase reporter gene (Promega). The pGL4.50[*luc2*/CMV/Hygro] vector (Promega) encoding the Firefly luciferase reporter gene *luc2* (*Photinus pyralis*) was used for transfection efficiency. *Escherichia coli* cells (MAX Efficiency DH5α Competent cells, Invitrogen) were transformed with the recombinant plasmid DNA and individual colonies were then screened for the presence of the DNA insert by PCR (*Taq* DNA Polymerase with ThermoPol Buffer, NEB). Positively identified clones were sent for Sanger sequencing analysis. NCBI BLAST confirmed the absence of mutations. Finally, the selected plasmid DNA clones were further expanded and purified (NucleoBond Xtra Midi Plus EF, Machery-Nagel).

The luciferase reporter containing the *BACH2* minimal promoter (−725; +146), pNL1.1/minP_BACH2_, was constructed through PCR amplification from tonsil B cell DNA as previously described by ref. ^41^, followed by NheI/XhoI restriction digestion and ligation into the pNL1.1 [Nluc] vector.

The luciferase reporter vector containing the 228 bp (+1265; +1493) *BACH2* enhancer (Enh), pNL1.1/minP_BACH2_/Enh, was constructed through PCR amplification from the PAC clone RP1-104D1 followed by XhoI restriction digestion and downstream ligation into the *BACH2* minimal promoter construct pNL1.1/minP_BACH2_. The enhancer was then sub-cloned from pNL1.1/minP_BACH2_/Enh and ligated into the XhoI site of the independent minimal promoter vector pNL3.1[*Nluc*/minP] to generate minP_PNL3.1_/Enh. All enhancer inserts were screened by PCR to identify sequence ligations in both the 5′-3′ and 3′-5′ orientation. Sequencing confirmed the orientation of inserts.

The remaining luciferase reporter constructs containing fragmented sequences of the *BACH2* enhancer, namely Enh80 (+1265/+1366), Enh122 (+1388/+1493) and Enh115 (+1265/+1381) were generated by PCR amplification with primers containing XhoI restriction sites at the 5′end and ligation into pNL1.1/minP_BACH2_. pNL1.1/minPBACH2/Δ21nt-Enh was generated by sub-cloning the Enh122 sequence by blunt ended ligation into the EcoRV site of the pNL1.1/minP_BACH2_/Enh80 vector. All fragmented enhancer inserts were screened by PCR to identify sequence ligations in both the native 5′-3′ and reverse 3′-5′ orientation.

For preparation of the mutant Mut-PU1bs and Mut-ELK1bs forms of the enhancer, pNL3.1/Enh was implemented as DNA template for PCR site-directed mutagenesis (Q5 Site-Directed Mutagenesis Kit, cat no. E0554 from NEB). The presence of the substitution mutations within the enhancer was confirmed by sequencing. The mutated enhancer sequences were then sub-cloned by XhoI restriction digestion into the XhoI site of pNL1.1/minP_BACH2_ to generate pNL1.1/minP_BACH2_/Mut-PU1bs and pNL1.1/minP_BACH2_/Mut-ELK1bs.

### Luciferase reporter assays

The following luciferase assay experiments were performed on primary naive B cells, transfected using the Amaxa Cell Line Nucleofector Kit V (cat no. VCA-1003 from Lonza) and cultured under the conditions of the in vitro B cell differentiation model. Depending on the day of electroporation between 1.0 and 5.0 × 10^6^ B cells were re-suspended in transfection buffer and combined with 4–5 μg of appropriate BACH2 containing NanoLuc reporter vectors together with 1 μg of control plasmid, pGL4.50[*luc2*/CMV/Hygro]. B cells were electroporated using program O-17 of the Amaxa Nucleofector II Device (Lonza), re-suspended with pre-incubated media and cultured at 37 °C for 24 h. 40% transfection efficiency was observed with 2 μg pmax-GFP and ~80–90% cell viability with 2–4 μg DNA. Cells were then collected, washed with PBS and centrifuged at 1800 r.p.m. (×2). Finally the cells were lysed in 50 μL 1X Passive Lysis Buffer (Promega) for 15 min at room temperature followed by storage of the lysate at −80 °C. The Nano-Glo Dual-Luciferase reporter Assay system (Promega) was used to detect luciferase expression from the NanoLuc and pGL4.50[*luc2*/CMV/Hygro] reporter vectors within lysate samples using the luminometer of the Varioskan Flash Multimode Reader (Thermo Scientific). The luciferase expression values, read as relative light units (RLU), from both reporter vectors were averaged and NanoLuc was normalised to firefly luciferase. Ratios of RLU were then normalised to that of control promoterless condition (pNL1.1) or minP_BACH2_ or minP_PNL3.1_.

For the kinetic experiments of pNL1.1/minP_BACH2_/Enh and pNL3.1/Enh, naive B cells were electroporated starting from D0 through to D5. D6 plasmablasts were initially electroporated on D4 and cell sorted as CD20^lo^CD38^hi^ cells on D6. For the kinetic experiment of pNL1.1/minP_BACH2_/Δ21nt-Enh, activated B cells were electroporated between D1-D4. Following each electroporation, transfected cells were placed back into the stimulation medium that corresponded with the two-step culture system and the luciferase activity of all the constructs was determined 24 h later (or 48 h for D6 plasmablasts).

To determine the effect of IL-2 on the *BACH2* enhancer in total activated B cells, cells were electroporated on D2 with pNL1.1/minP_BACH2_/Enh (native orientation) and subsequently placed back into culture with or without IL-2 for 24 h. For identification of the sub-population of dividing and proliferating cells, naive B cells stained with CFSE on D0 were electroporated on D2 with pNL1.1/minP_BACH2_/Enh (native orientation) and cultured for 24 h and 48 h. On D3, cells were stained with Hoechst and sorted into three populations CFSE^hi^H^−^, CFSE^hi^H^+^ and CFSE^lo^. On D4, cells were sorted on CFSE staining alone into CFSE^hi^ and CFSE^lo^ populations.

### RNA-seq

Three experiments from independent blood donors were analysed. RNA extractions were performed with the NucleoSpin RNA XS kit (cat no. 740902.250 from Macherey-Nagel). QC, library preparation and sequencing were performed by Helixio company (Clermont Ferrand, France). Briefly, libraries were prepared with the TruSeq Stranded mRNA Library Prep Kit (cat no. RS-122-2101 from Illumina) and samples were sequenced on an Illumina NextSeq 500 using 75-bp single-end reads (NextSeq 500 High Output v2, Illumina). Quality of sequencing data was monitored by FastQC. Residual adapters from sequencing were trimmed using cutadapt 1.0. Potential PCR duplicates were removed using SAMtools 1.3. Reads were then aligned on the GRCh38 human genome using STAR 2.4.2a. Differential expression on filtered genes (HTSfilter 1.7.1), were performed using DESeq2 in R 3.3.1. Genes were declared differentially expressed with a false discovery rate < 5%. For heatmap, genes differentially expressed with fold change >1.4 in CD25^hi^ and siBACH2 condition compare to CFSE^lo^ population was ploted. Log2 normalised read counts from DESeq2 were used to generate the representation with gplots 3.0.1, and RColorBrewer 1.1–2. Hierarchical clustering was generated using Euclidean distance matrix with complete linkage.

### ChIP-seq

BACH2 antibody performance used for the ChIP (D3T3G, Cell signalling) was certified by Active Motif Epigenetic Services. Activated naive B cells at D3 of the culture were fixed with 1% formaldehyde for 15 min at room temperature. Cross-linking was quenched with Glycerine. For immunoprecipitation, 28 μg of chromatin and 40 μL of antibody were used. Control, siBACH2 and input were sequenced and aligned on hg19. For comparative analysis, standard normalisation is achieved by down-sampling the usable number of tags for each sample in a group to the level of the sample in the group with the fewest usable number of tags. Peaks were called using the MACS 2.1.0 algorithms^69^. MACS default cutoff is p-value 1e-7 for narrow peaks and 1e-1 for broad peaks. Peak filtering was performed by removing false ChIP-seq peaks as defined within the ENCODE blacklist^70^.

Known motifs were identified with the findMotifsGenome program of the HOMER package^71^ using default parameters and input sequences comprising ±100 bp from the centre of the top 1000 peaks.

BACH2 target analysis was realized using BETA^72^ using the default parameters with differential gene expression data set (siBACH2 vs. uncommitted B cells, *p* < 0.05) and ChIP-seq data.

### Statistical analyses

GraphPad Prism software was used for statistical analysis using the Mann−Whitney non-parametric test or the two-tailed unpaired Student’s *t*-test if not stated otherwise.

### Data availability

The data supporting the findings of this study are available within the article and its Supplementary Information files, or are available on reasonable request from the corresponding authors. RNA-seq and ChIP-seq data have been deposited in Gene Expression Omnibus with the primary accession code GSE102460.

## Electronic supplementary material

Supplementary Information

Description of Additional Supplementary Files

Supplementary Data 1

Supplementary Data 2
