# Supplementary material for: IL-2 imprints human naive B cell fate towards plasma cell through ERK/ELK1-mediated BACH2 repression
Source: Nat Commun. 2017 Nov 13;8:1443. doi: 10.1038/s41467-017-01475-7 (PMC5682283; doi:10.1038/s41467-017-01475-7)
Supplement: Supplementary file 1 — Supplementary Information [file 41467_2017_1475_MOESM1_ESM.pdf]

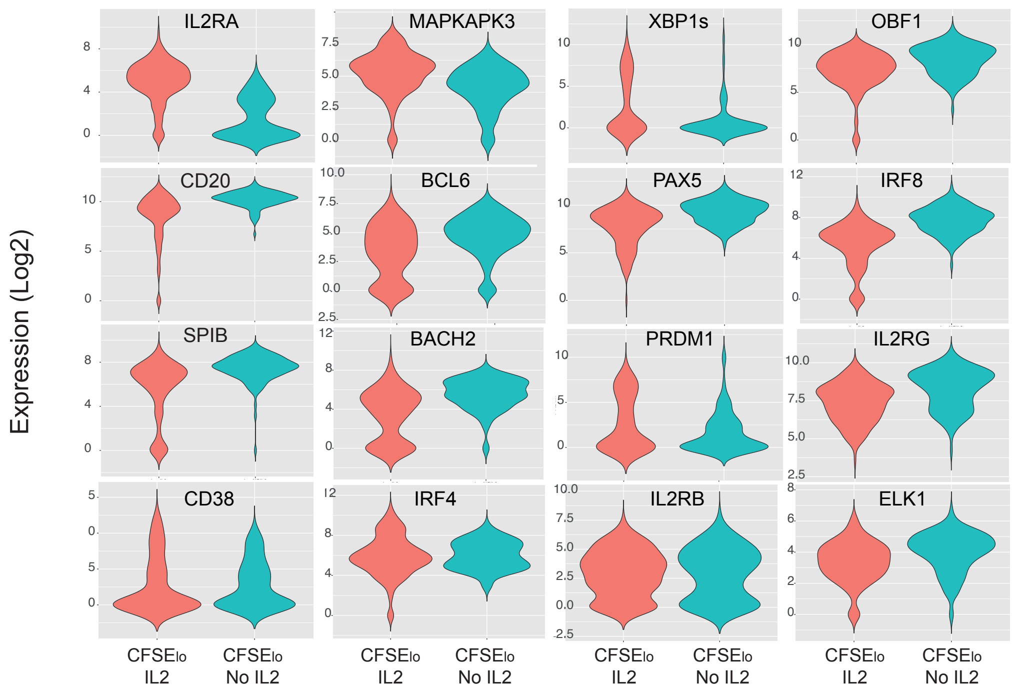

Supplementary Figure 1: Comparison of gene expression levels analysed by single-cell QRT-PCR between D4 CFSE^{lo} cells when primed or not with IL-2.

Violin plots of gene expression: in blue the D4 CFSE^{lo} cells (CFSE$_{lo}$ No IL2, n=110), and in red the IL-2-primed counterpart (CFSE$_{lo}$ IL2, n=175). Expression (vertical axis) is the log2-transformed fold change over median gene expression level for each condition. Width of the violin indicates frequency at the expression level. Genes are ranked by their ANOVA p-value calculated with PRISM: *IL2RA, XBP1s, OBF1, CD20, BCL6, IRF8, SPIB, PAX5, MAPKAPK3, BACH2* p< 10^{-4}; *PRDM1* p<10^{-3}; *IL2RG* p<0.005; *CD38, IRF4, IL2RB, ELK1* non-significant (ANOVA).

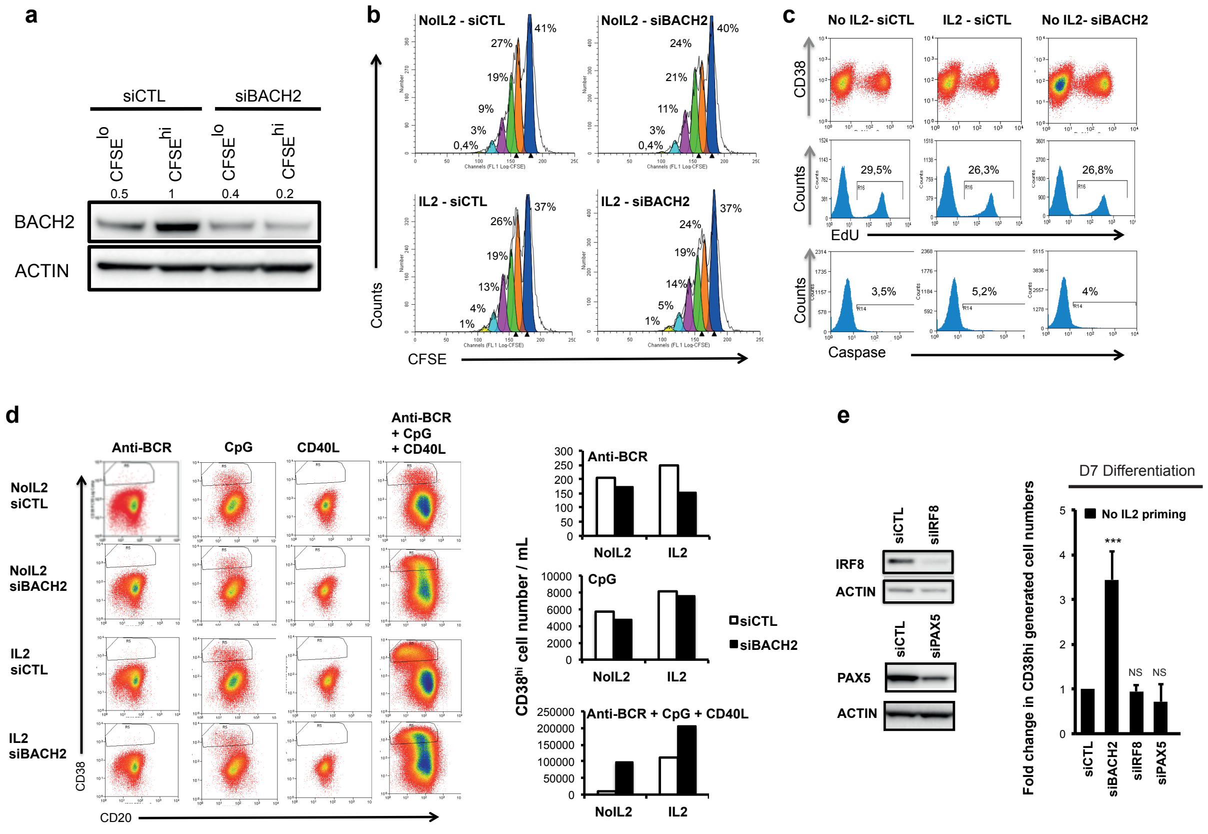

Supplementary Figure 2: Plasma cell differentiation mechanism triggered by BACH2 inhibition is independent of a proliferation or precocious differentiation effect and remains dependent of the combined BCR, TLR9 and CD40 signaling activation.

(**a**) The efficiency of BACH2 inhibition with siRNA (siBACH2) was monitored at D4, 48h after cell electroporation in sorted populations based on CFSE dilution. Non-targeting siRNA (siCTL) was used as control. Values are the average fold changes in the expression levels of *BACH2* normalized to β-ACTIN levels for two independent experiments. (**b**) Naive B cells were CFSE stained and activated for 2 days before electroporation with siBACH2 or a negative control (siCTL), then stimulated with or without IL-2. CFSE dilution was assessed at D4 by flow cytometry and analysed with ModFit. One experiment representative of three is shown. (**c**) Flow cytometry analyses of D4-activated B cells that were stimulated or not with IL-2 and electroporated with siBACH2 or siCTL at D2. Proliferation was assessed by EdU staining and survival by active caspase 3 staining. CD38 staining does not reveal premature differentiation of B cells deficient for BACH2. (**d**) D7 flow cytometry analysis of CD38 up regulation by B cells that were activated with anti-BCR alone, CpG or CD40L (conditions that do not enable B cell proliferation) or the cocktail of the three stimuli sufficient to trigger proliferation. At D2, cells were electroporated with siBACH2 or siCTL and stimulated with or without IL-2 (No IL2). Absolute CD38^{hi} cell number generated at D7 depending on the activation cocktail (anti-BCR or CpG or anti-BCR+CpG+CD40L), the IL-2 priming, and BACH2 inhibition (siBACH2) is shown for one experiment representative of two. (**e**) Efficiency of IRF8 and PAX5 inhibition on protein expression levels at D4, 48h after electroporation, analysed by immunoblotting. β-Actin was used as loading control and levels of protein expression in siCTL electroporated cells were used as references. Plasma cell differentiation induced by siRNA electroporation of activated B cells at D2 and monitored by flow cytometry at D7. Data are normalized to CD38^{hi} cell number generated by the control cells (siCTL). siIRF8 (n=4), siPAX5 (n=3), siBACH2 (n=7). Significant differences compared with CD38^{hi} cell number in the reference condition (IL2-siCTL) are shown, ***p<0.001, NS: No significant differences (t-test).

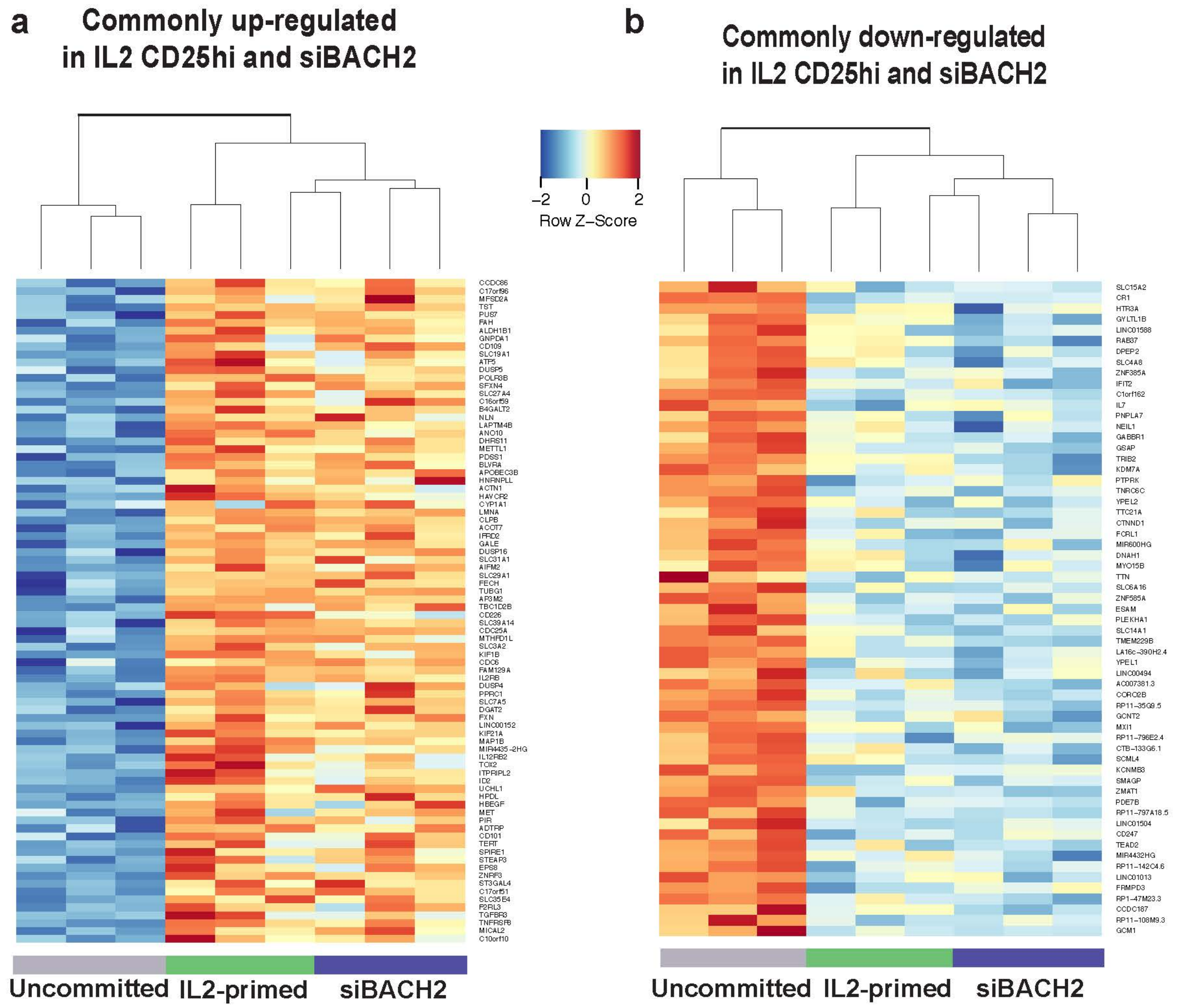

Supplementary Figure 3: Transcriptomic analysis reveals a common signature shared between IL-2 committed cells and siBACH2 committed cells.
(**a**) Expression profile of the 84 up-regulated genes shared between D4 $CFSE^{lo}CD25^{hi}$ cells (IL2-primed) and D4 $CFSE^{lo}$ siBACH2 cells (siBACH2) compared with D4 $CFSE^{lo}$ control cells (uncommitted), FC>1.4 and p<0.05 (Wald test), n=3 for each condition. (**b**) Expression profile of the 61 down-regulated genes shared between D4 $CFSE^{lo}$ $CD25^{hi}$ cells (IL2-primed) and D4 $CFSE^{lo}$ siBACH2 cells (siBACH2) compared with D4 $CFSE^{lo}$ control cells (uncommitted), FC>1.4 and p<0.05 (Wald test), n=3 for each condition.

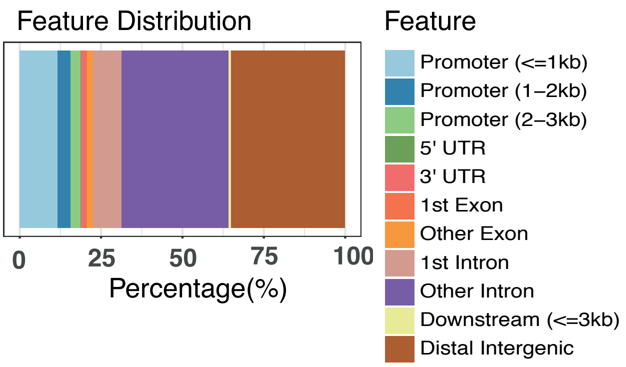

Supplementary Figure 4: BACH2 ChIP-seq analysis.
Relationship of genome-wide BACH2 binding sites to annotated genes in D3 activated B cells in absence of IL-2.

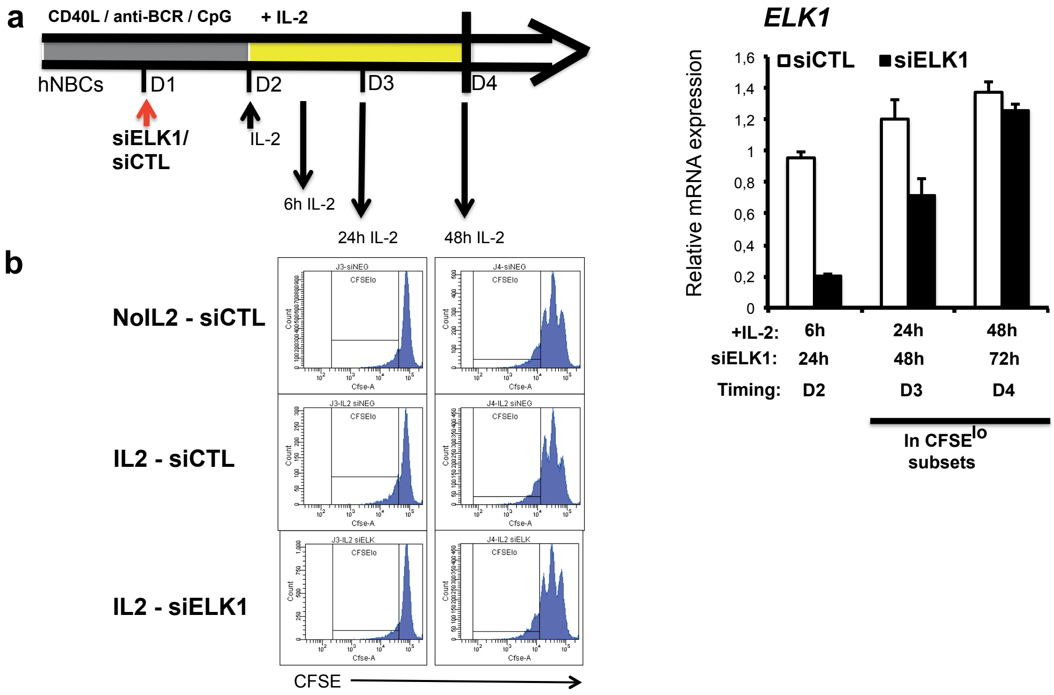

Supplementary Figure 5: ELK1 inhibition does not affect cell proliferation.
(**a**) Expression level of *ELK1* analysed by QRT-PCR at different time points of the activation process of naive B cells (hNBCs) electroporated at D1 by siELK1 or the non-targeting control siRNA (siCTL). One experiment representative of three, the s.d. is shown. Further *BACH2* expression analysis was performed 48h after siELK1 electroporation (related to Fig. 7b). (**b**) Effect of ELK1 inhibition on CFSE dilution analysed by flow cytometry at D3 and D4. Naive B cells were electroporated at D1 with siELK1 or the negative control siCTL. One representative experiment out of 4 is shown.

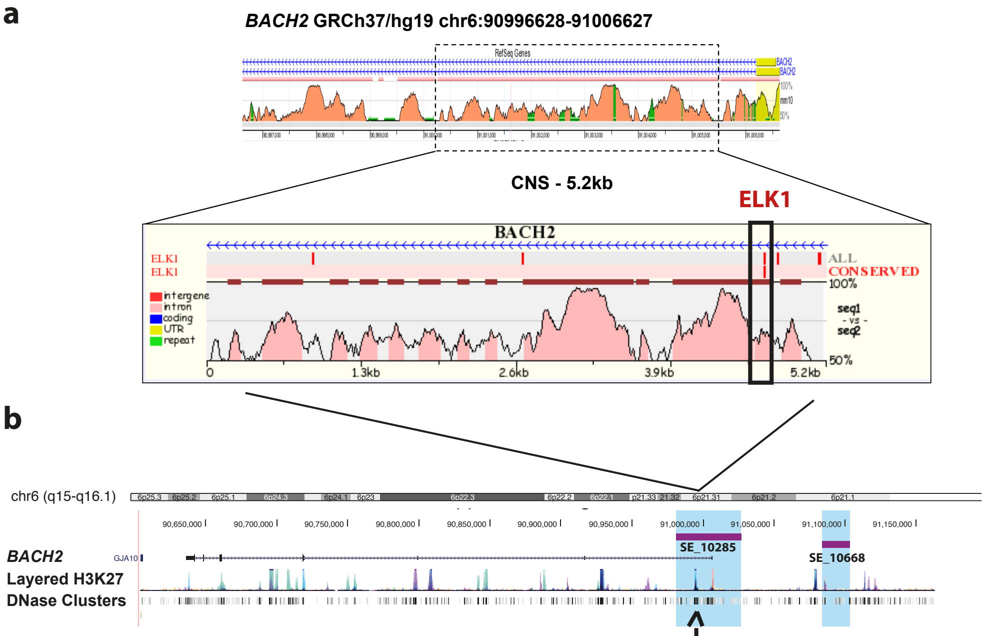

Constituent ID	SE ID	Chrom	Start	End	Constituent Size	MACS score
C092786	SE_10285	chr6	90981257	90985749	4492	3100
C092787	SE_10285	chr6	90993591	90997203	3612	3100
C092788	SE_10285	chr6	91003598	91008570	4972	3100
C092789	SE_10285	chr6	91012572	91015879	3307	3100
C092790	SE_10285	chr6	91021038	91026895	5857	3100

Constituent ID	SE ID	Chrom	Start	End	Constituent Size	MACS score
C092791	SE_10668	chr6	91084014	91087417	3403	3100
C092792	SE_10668	chr6	91091287	91096510	5223	3100
C092793	SE_10668	chr6	91102487	91103503	1016	234

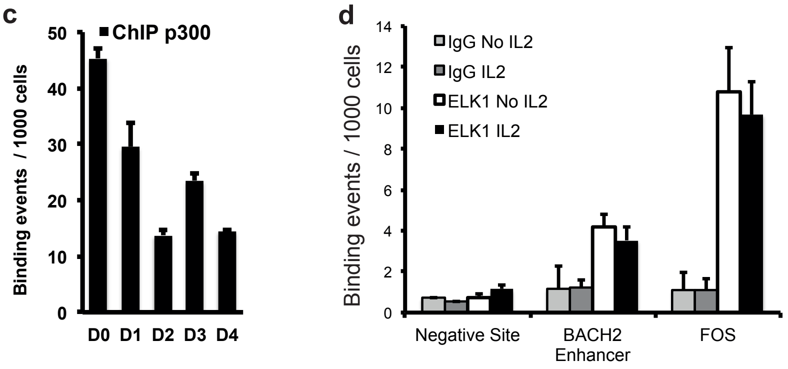

Supplementary Figure 6: Characterisation of an active regulatory region within the *BACH2* super-enhancer.
(a) VISTA plot of conserved non-coding sequences (CNSs) adjacent to the *BACH2* transcription start site. A 5.2kb CNS within intron 1 is conserved between human and mice *BACH2* locus. Out of the 5 putative ELK-1 binding sites identified using Transfac only the site of interest is conserved with mice (rVISTA) as shown by the 10bp sequence alignment (Jalview). **(b)** Alignments showing mapped *BACH2* super enhancer (SE), H3K27ac from 7 cell lines from ENCODE and clusters of DNAse I hypersensitivity (from 125 lines ENCODE). Arrows indicate the localisation of the conserved ELK1 binding site. Super-enhancer positions were obtained using CD19 primary data from *Hnisz D. et al., Cell 2013*, and analysed with dbSUPER. **(c)** ChIP analysis of p300 binding on *BACH2* enhancer during activation of naive B cells *in vitro*. One representative experiment is shown with the s.d. (n=3). **(d)** ChIP assay of the *in vivo* binding of ELK1 in D3 B cells primed or not with IL-2. DNA immunoprecipitated by total ELK1 antibody or isotype control (IgG) was amplified by real time PCR using primers flanking the putative ELK1 binding site in *BACH2* intron 1 (BACH2 enhancer), the known ELK1 binding regions in *FOS* gene and negative control primers as a reference (negative site). Average with the s.d. of three independent experiments is shown.

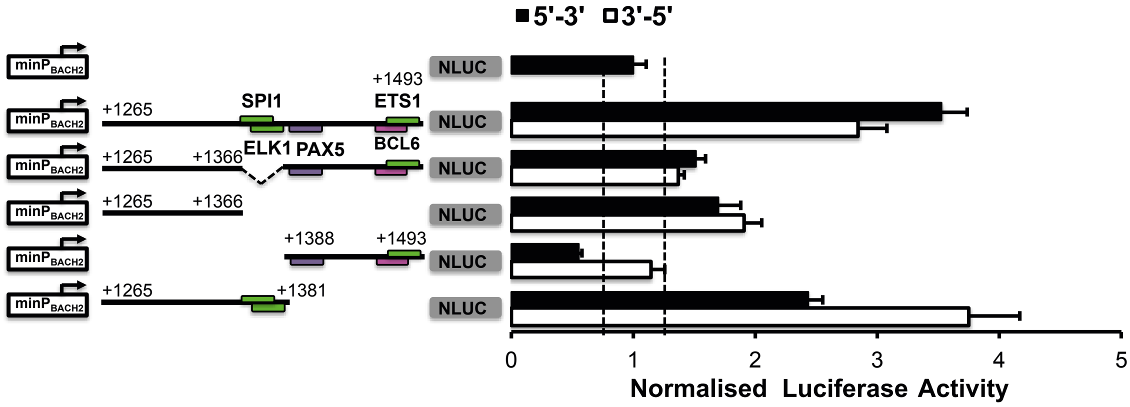

Supplementary Figure 7: Regulatory elements analysis in the 228nt enhancer sequence. Candidate binding motifs predicted by MATINSPECTOR in the enhancer sequence are shown (green boxes: ETS1 family members; purple box: PAX5; pink box: BCL6). Luciferase activity of the *BACH2* promoter alone (line 1), full-length enhancer sequence (line 2), and ELK1 deleted fragments with deletions from the 5' end or 3' end. Numbers adjacent to diagrams indicate positions relative to the transcription start site. Fragments were systematically cloned in both orientations (5'-3' and 3'-5') in the luciferase reporter plasmids, downstream from the proximal BACH2 promoter ($minP_{BACH2}$). Activated B cells were electroporated with the reporter constructs on D2 and cultured 24h in presence of IL-2. Luciferase activity (mean +/- s.e.m. from 2 to 12 independent experiments) is presented relative to *BACH2* promoter activity alone (line 1). Statistically significant cutoff values (dotted lines) were obtained by adding 2 standard deviations to the mean value obtained for the promoter activity. Deletion of the ELK1 binding motif (positions +1367 to +1387, line 3) resulted in diminished luciferase activity almost equal to that of the $minP_{BACH2}$. In support of a positive role of ELK1 motif, other constructs with deletions of the ELK1 binding motif (+1265 ; +1366, line 4) and (+1388 ; +1493, line 5) also resulted in lower transcriptional activity, while the truncated enhancer sequence which carries the ELK1 motif (+1265 ; +1381, line 6) recapitulated activity of the full-length enhancer. In summary, the ELK1 motif appeared to be a major positive regulatory element at this time point of human B cell activation. Other minor regulatory elements, activator (+1265 ; +1366) or repressor (+1388 ;+1493), could be involved in the enhancer function.

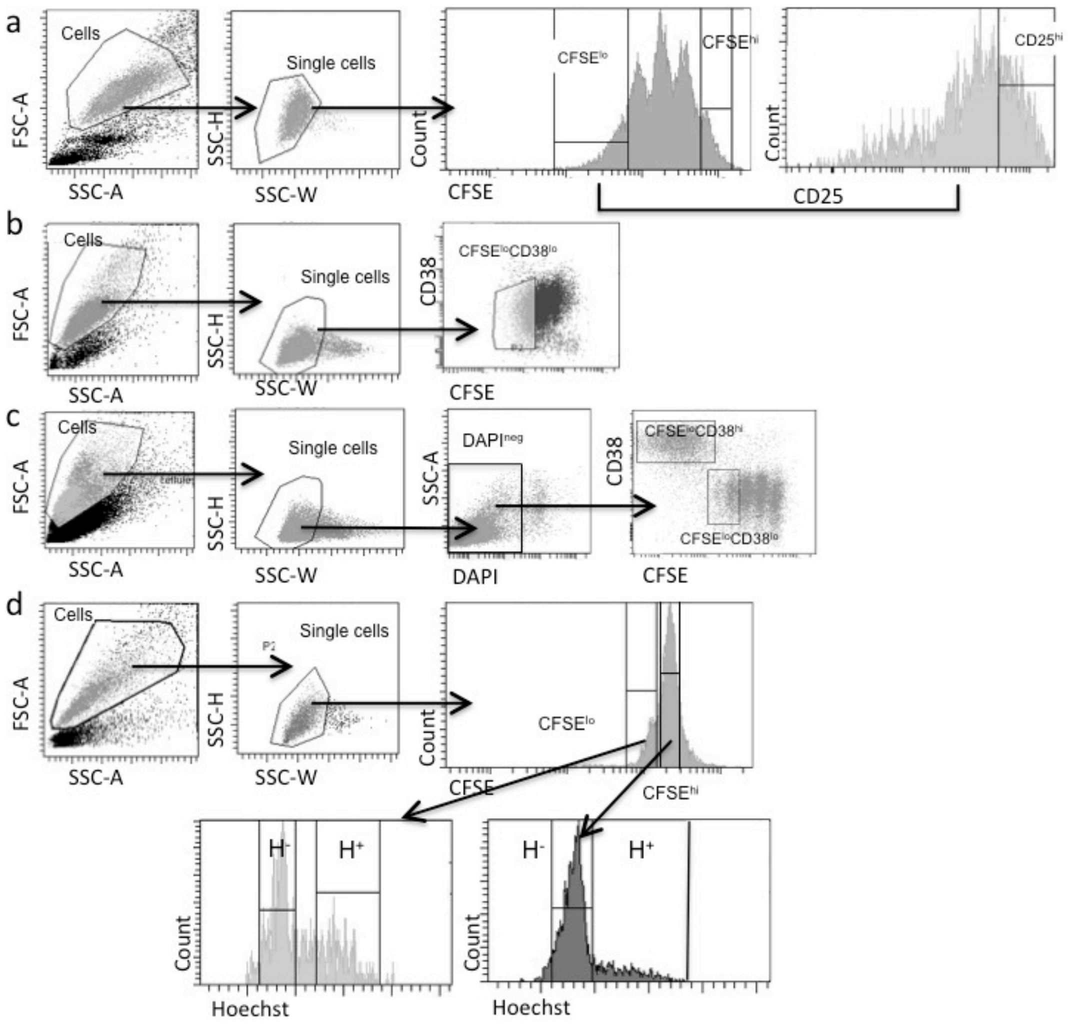

Supplementary Figure 8: Gating strategies for flow cytometry analyses and cell sorting.
Naive B cells were stained with CFSE at D0 and activated *in vitro*. (**a**) Gating strategy to sort CFSE^{hi} activated B cells at D4 (related to Fig. 9g and Fig.10b), the CFSE^{lo} population related to Fig. 1c, Fig. 2a and Fig.10b , and CFSE^{lo}CD25^{hi} cells related to Fig. 1d-e. (**b**) Gating strategy to sort D5 CFSE^{lo}CD38^{lo} population related to Fig. 2a. (**c**) Gating strategy to sort D7 CFSE^{lo}CD38^{hi} and CFSE^{lo}CD38^{lo} populations related to Fig. 2a-b and Fig. 3c-e. (**d**) Gating strategy to sort D3 CFSE^{hi}Hoechst^{neg}, CFSE^{lo}Hoechst^{+} and CFSE^{lo}Hoechst^{neg} populations related to Fig. 3c and Fig. 9f.

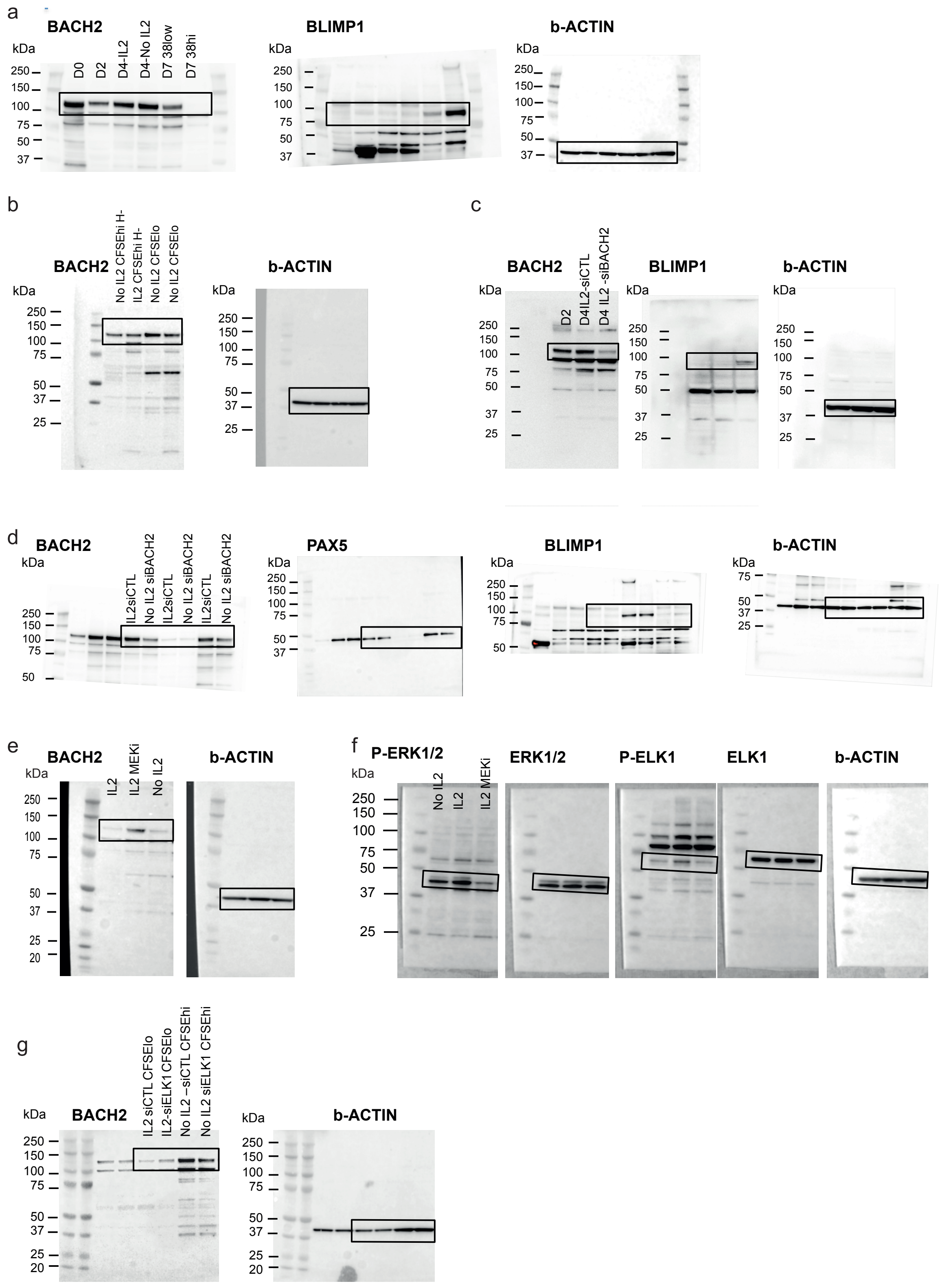

Supplementary Figure 9: Uncropped original images of immunoblots.

(**a**) Immunoblot data corresponding to Fig. 2b. (**b**) Immunoblot data corresponding to Fig. 2c. (**c**) Immunoblot data corresponding to Fig. 2d. (**d**) Immunoblot data corresponding to Fig. 3e. (**e**) Immunoblot data corresponding to Fig. 4a. (**f**) Immunoblot data corresponding to Fig. 7a. (**g**) Immunoblot data corresponding to Fig. 10b. Data cropped from the image are cycled in rectangles.

Supplementary Table 1: List of antibodies used for the flow cytometry analysis.

Antibody	Clone	Isotype	Source	Dilution
CD27-PE	1A4CD27	Mouse IgG1	Beckman Coulter	7/100
CD19-FITC	J3-119	Mouse IgG1	Beckman Coulter	7/100
CD20-FITC	B9E9	Mouse IgG2a	Beckman Coulter	7/100
CD38-PC5.5	LS198-4-5	Mouse IgG1	Beckman Coulter	3/100
CD138-PE	B-A38	Mouse IgG1	Beckman Coulter	1/11
IgM-FITC	G20-127	Mouse IgG1	BD Biosciences	1/11
IgG-PE	G18-145	Mouse IgG1	BD Biosciences	1/11
STAT5 PY694	47	Mouse IgG1	BD Biosciences	1/11
PAX5	REA140	REA control1	Miltenyi Biotec	1/11
IRF8	REA516	REA control1	Miltenyi Biotec	1/11
IRF4	REA201	REA control1	Miltenyi Biotec	1/11
BLIMP1	IC36081A	Mouse IgG1	R&D Systems	1/11

Supplementary Table 2: List of TaqMan primers used for QRT-PCR.

TaqMan Primer	Reference
SOCS2	*Hs00919620_m1*
CISH	*Hs00367082_g1*
MAPKAPK3	*Hs00177957_m1*
IL2RA	*Hs00907779_m1*
IL2RB	*Hs00168402_m1*
IL2RG	*Hs00953624_m1*
IRF8	*Hs00175238_m1*
BACH2	*Hs00222364_m1*
XBP1s	*Hs03929085_g1*
PRDM1	*Hs00153357_m1*
IRF4	*Hs00180031_m1*
BCL6	*Hs00153368_m1*
PAX5	*Hs00277134_m1*
SPIB	*Hs00162150_m1*
ELK-1	*Hs00901847_m1*
MYD88	*Hs01573837_g1*
CD19	*Hs00174333_m1*
CD20 (MS4A1)	*Hs00544819_m1*
CD27	*Hs00386811_m1*
CD38	*Hs01120071_m1*
CD138 (SDC1)	*Hs00896423_m1*
HPRT1	*Hs02800695_m1*

Supplementary Table 3: List of antibodies used for western blotting.

Antibody	Clone	Isotype	Source	Dilution
IRF8	D20D8	Rabbit IgG	Cell Signaling	1/1000
BACH2	Polyclonal	Rabbit	Abcam	1/1000
PAX5	3852-1	Rabbit IgG	Abcam	1/1000
BCL6	EP529Y	Rabbit IgG	Abcam	1/1000
p44/42	137F5	Rabbit IgG	Cell Signaling	1/1000
Phospho-p44/42 (T202/Y204)	D13.14.4E	Rabbit IgG	Cell Signaling	1/1000
ELK-1	E277	Rabbit IgG	Abcam	1/1000
Phospho-ELK-1(S383)	B-4	Mouse IgG	Santa Cruz	1/1000
STAT5	89	IgG2b	Biosciences	1/1000
Phospho-STAT5(PY694)	47	Mouse IgG1	BD Biosciences	1/1000
GAPDH	14C10	Rabbit IgG	Cell Signaling	1/2000
β-Actin	AC15	Mouse IgG1	SIGMA	1/2000

Supplementary Table 4: List of primers used for ChIP-QPCR.

Gene locus	Forward Sequence (5'->3')	Reverse Sequence (5'->3')
BACH2 promoter	GAAAACGCTAGCTAACCCTGG	CATAGCAGGGTGAGAGTAGTGG
BACH2 enhancer	ATCCCCAGCACTCTCCCGCG	CAGCGGCCGCGCCGGAACTTC
MCL1	AGTCCCCAACTATGCCCTCT	CTCTGTGCTTCCCTGAGACC
MYD88	GCCGAGGCTCTAATTCCTCT	CCCCGCATGTCTGTAAAATC
FOS	GAGCAGTTCCCGTCAATCC	GCATTTCGCAGTTCCTGTCT

Supplementary Table 5: List of siRNAs.

siRNA	Source	Reference
siELK-1	Thermo Scientific	L-003885-00
siBACH2	Thermo Scientific	L-009787-00
siBCL6	Thermo Scientific	L-011591-01
siPAX5	Thermo Scientific	L-012241-00
siIRF8	Thermo Scientific	L-011699-00
siCTL	Thermo Scientific	D-001810-10

Supplementary Table 6: List of cloning and sequencing primers used for luciferase reporter construction.

Fragment Name	Forward Sequence (5'->3')	Reverse Sequence (5'->3')
$minP_{BACH2}$ (-725; +146)	GTGGAGCATCTTTTCTTGCCCTT	CTCGCTCGAGAACTTTGCGTCCTTTT
Enh (+1265; +1493)	ATACTCGAGGAGTGAGTGTGTGAAGGGGT	TTTCTCGAGGTCCCTCTCGTTTCCTGGAG
Enh80 (+1265/+1366)	ATACTCGAGGAGTGAGTGTGTGAAGGGGT	CGCGCTCGAGAAGTTACCAAACTCGCCTACG
Enh115 (+1265/+1381)	ATACTCGAGGAGTGAGTGTGTGAAGGGGT	TTTCTCGAGCCGGAACTTCCCTCAAGTTA
Enh122 (+1388/+1493)	CGGCTCGAGCTGCCGGGGTGGTTTG	TTTCTCGAGGTCCCTCTCGTTTCCTGGAG
Mut-PU1bs	GTAACTTGAGaaaaGTTCCGGCGCG	CAAACTCGCCTACGCGCG
Mut-ELK1bs	TTGAGGGAAGttttGGCGCGGCCG	GTTACCAAACTCGCCTACGC
