# Supplementary material for: IL-2 imprints human naive B cell fate towards plasma cell through ERK/ELK1-mediated BACH2 repression
Source: Nat Commun. 2017 Nov 13;8:1443. doi: 10.1038/s41467-017-01475-7 (PMC5682283; doi:10.1038/s41467-017-01475-7)
Supplement: Supplementary file 2 — Description of Additional Supplementary Files [file 41467_2017_1475_MOESM2_ESM.pdf]

Description of Additional Supplementary Files

File Name: Supplementary Data 1
Description: RNA-seq analyses of differentially expressed genes between D4-committed cells and the uncommitted counterpart. Number of genes refers to the Venn analyses (Fig. 5a).

File Name: Supplementary Data 2
Description: Lists of genes up regulated in D4 CFSElo B-cells deficient for BACH2 ($p<0.05$, Wald test) and predicted to be direct targets of BACH2 by ChIP-seq analysis. These genes refer to the pieCHART analysis in Fig. 6e.
